# Evidence for an Independent Hydrogenosome-to-Mitosome Transition in the CL3 Lineage of Fornicates

**DOI:** 10.3389/fmicb.2022.866459

**Published:** 2022-05-19

**Authors:** Romana Vargová, Pavla Hanousková, Jana Salamonová, David Žihala, Jeffrey D. Silberman, Marek Eliáš, Ivan Čepička

**Affiliations:** ^1^Department of Biology and Ecology, Faculty of Science, University of Ostrava, Ostrava, Czechia; ^2^Department of Zoology, Faculty of Science, Charles University, Prague, Czechia; ^3^Department of Biological Sciences, University of Arkansas, Fayetteville, AR, United States; ^4^Institute of Parasitology, Biology Centre, Czech Academy of Sciences, České Budějovice, Czechia

**Keywords:** Caviomonadidae, caviomonads, codon reassignment, Fornicata, hydrogenosome, mitochondrial evolution, mitosome

## Abstract

Fornicata, a lineage of a broader and ancient anaerobic eukaryotic clade Metamonada, contains diverse taxa that are ideally suited for evolutionary studies addressing various fundamental biological questions, such as the evolutionary trajectory of mitochondrion-related organelles (MROs), the transition between free-living and endobiotic lifestyles, and the derivation of alternative genetic codes. To this end, we conducted detailed microscopic and transcriptome analyses in a poorly documented strain of an anaerobic free-living marine flagellate, PCS, in the so-called CL3 fornicate lineage. Fortuitously, we discovered that the original culture contained two morphologically similar and closely related CL3 representatives, which doubles the taxon representation within this lineage. We obtained a monoeukaryotic culture of one of them and formally describe it as a new member of the family Caviomonadidae, *Euthynema mutabile* gen. et sp. nov. In contrast to previously studied caviomonads, the endobiotic *Caviomonas mobilis* and *Iotanema spirale*, *E. mutabile* possesses an ultrastructurally discernible MRO. We sequenced and assembled the transcriptome of *E. mutabile*, and by sequence subtraction, obtained transcriptome data from the other CL3 clade representative present in the original PCS culture, denoted PCS-ghost. Transcriptome analyses showed that the reassignment of only one of the UAR stop codons to encode Gln previously reported from *I. spirale* does not extend to its free-living relatives and is likely due to a unique amino acid substitution in *I. spirale*’s eRF1 protein domain responsible for termination codon recognition. The backbone fornicate phylogeny was robustly resolved in a phylogenomic analysis, with the CL3 clade amongst the earliest branching lineages. Metabolic and MRO functional reconstructions of CL3 clade members revealed that all three, including *I. spirale*, encode homologs of key components of the mitochondrial protein import apparatus and the ISC pathway, indicating the presence of a MRO in all of them. *In silico* evidence indicates that the organelles of *E. mutabile* and PCS-ghost host ATP and H_2_ production, unlike the cryptic MRO of *I. spirale*. These data suggest that the CL3 clade has experienced a hydrogenosome-to-mitosome transition independent from that previously documented for the lineage leading to *Giardia*.

## Introduction

Reduction is a general evolutionary trend of endosymbiotic organelles. Speaking specifically about the mitochondrion, it can be inferred that massive reduction occurred along the path from the original α-proteobacterial endosymbiont (protomitochondrion) to the mitochondrial cenancestor, i.e., the mitochondrion in the last eukaryotic common ancestor (LECA; Roger et al., 2017). This was followed by further simplification during the post-LECA radiation of eukaryotes, with the observed pattern including recurrent loss of the same mitochondrial components and different degrees of reduction in different lineages (Janouškovec et al., 2017; Maciszewski and Karnkowska, 2019). While this general scheme is clearly established, its details remain to be worked out. For example, it seems that the mitochondrion of the LECA was more “bacterial” than commonly thought, as continued exploration of mitochondria of diverse protists is expanding the list of presumably ancestral bacterial traits of the organelle preserved in various limited subsets of eukaryotic lineages (Leger et al., 2015; Gray et al., 2020; Horváthová et al., 2021; Pyrih et al., 2021). On the opposite extreme, a number of eukaryote lineages were found to possess mitochondria that are to a varying degree simplified as compared to the textbook model of this organelle (reviewed by Roger et al., 2017), yet the list is certainly not complete, as additional mitochondrial variants are continually being reported from diverse corners of the eukaryote phylogeny (John et al., 2019; Lewis et al., 2020; Rotterová et al., 2020; Yahalomi et al., 2020).

The evolutionary trajectories followed by mitochondria in various lineages may result in transmogrification of the organelles to such a degree as making them hardly recognizable as mitochondrial, let alone bacterial, derivatives. One such category of mitochondrion-related organelles (MROs), the hydrogenosome, was discovered and studied for many years without the researchers realizing that it is in fact a special form of the mitochondrion in the respective organisms, which were instead thought to be (primitively) amitochondriate (Müller, 2007). Other putatively amitochondriate taxa eventually turned out to harbor another MRO form, the so-called mitosome, which is so reduced as to be essentially indiscernible by conventional cytological investigations, and its discovery required targeted searches for cellular structures containing homologs of common nucleus-encoded mitochondrial proteins (Mai et al., 1999; Williams et al., 2002; Tovar et al., 2003). The finding of reduced mitochondrial derivatives in taxa previously considered to be amitochondriate was a crucial step toward the currently favored view that all extant eukaryotes evolved from a mitochondrion-bearing ancestor (Roger et al., 2017). Nevertheless, one eukaryote lineage, the oxymonads, has been confirmed to include genuine amitochondriates, resulting from a secondary loss of the organelle (Karnkowska et al., 2016). Whether the complete loss of the mitochondrion is restricted to this single case or whether additional amitochondriate eukaryotes may exist is presently unknown.

The multiple independent origins of hydrogenosomes, mitosomes, and other MROs forms that defy simple classification is the result of convergence in mitochondrial evolution. The primary driving force behind such evolutionary transformations is the adoption of microaerophilic or anaerobic lifestyles (Stairs et al., 2015). As a result, MROs are characterized by having dispensed with the canonical mitochondrial functional pathways and processes related to aerobic metabolism, such as the Krebs cycle, the respiratory chain and ATP production via harnessing an electrochemical potential across the inner mitochondrial membrane. These organelles often lack their own genome and associated systems of gene expression (Roger et al., 2017). The transition to obligate anaerobiosis in eukaryotes is a reoccurring phenomenon, with some lineages having undergone it rather recently while others representing deeply diverged species-rich clades. Accordingly, MROs comprise a wide spectrum of forms differing in their degree of reduction and transformation (Maguire and Richards, 2014; Roger et al., 2017). For example, while hydrogenosomes by definition still function as energetic organelles that supply the cell with ATP formed by substrate-level phosphorylation, mitosomes do not produce ATP.

Current data indicate that the most ancient anaerobic eukaryote lineage is the Metamonada, an assemblage of flagellates robustly resolved as a monophyletic group but with an uncertain placement in the eukaryote phylogenetic tree (Karnkowska et al., 2016; Burki et al., 2020). Metamonads were divided into three subclades, each defined both by a unique suite of ultrastructural characters and molecular phylogenetic evidence: Preaxostyla, Parabasalia, and Fornicata (Adl et al., 2019). Each of these groups evolved from and contains free-living forms, but they are currently dominated by endobiotic taxa living in anoxic niches provided by animal bodies. Recently, two more metamonad lineages, represented by the poorly studied free-living barthelonids (*Barthelona* and its formally undescribed relatives) and anaeramoebids (presently equal to the genus *Anaeramoeba*), have been discovered (Yazaki et al., 2020; Stairs et al., 2021). Each of these lineages, especially anaeramoebids, has been instrumental in illuminating the nature of the MRO ancestral for metamonads: this is inferred to have been a genome-lacking organelle possessing functions typical for the hydrogenosome, i.e., production of H_2_ as a metabolic waste product related to the generation of ATP, but with additional physiological functions typical of the classical mitochondrion, such as a disulfide relay system, propionate production, and amino acid metabolism (Stairs et al., 2021). Despite this recent re-evaluation of the ancestral complexity of the metamonad MRO, it still represents a massive mitochondrial reduction and transformation along the metamonad stem lineage leading to the radiation of the different extant metamonad subgroups, which is estimated to date back to at least one billion years ago (Parfrey et al., 2011).

This vast time span has allowed for a lot of evolutionary experimentation with the ancestral metamonad MRO, resulting in a high diversity of MRO forms in extant metamonads (Jerlström-Hultqvist et al., 2013; Zubáčová et al., 2013; Leger et al., 2017; Yazaki et al., 2020; Stairs et al., 2021). Thus, some members, including *Anaeramoeba*, Parabasalia, and some lineages of the Fornicata, have retained hydrogenosomes. Other taxa, such as the preaxostylan *Paratrimastix pyriformis*, the fornicate *Dysnectes brevis*, and the single barthelonid investigated in this regard, exhibit MROs that may produce H_2_ without a link to ATP generation. Such a MRO type has most recently been hypothesized to also occur in the so-called vertebrate retortamonads, a sister group of diplomonads within Fornicata (Füssy et al., 2021; Stairs et al., 2021), and seems to have been preserved in some diplomonads (e.g., the free-living *Trepomonas*; Leger et al., 2017). However, other diplomonads exhibit typical hydrogenosomes that are thought to have evolved by secondarily acquiring MRO-localized ATP production via horizontal gene transfer (e.g., the endobiotic *Spironucleus salmonicida*) or, as is the case of the *Giardia* lineage, have further reduced the MRO into a mitosome lacking both H_2_ and ATP production. Finally, complete MRO loss has occurred in oxymonads (in at least one species but potentially in the whole group; Karnkowska et al., 2016, 2019).

Despite the recent progress in mapping the diversity and evolutionary history of the mitochondrion in metamonads, the current picture is still to a large extent provisional and incomplete. One of the metamonad lineages whose mitochondria have not yet been investigated are the caviomonads (the family Caviomonadidae). The eponymous representative, *Caviomonas mobilis*, is a small anaerobic flagellate found in the intestinal tract of rodents (Nie, 1950). The single transmission electron microscopy (TEM) study of this organism did not reveal any candidate for a mitochondrion or a MRO (Brugerolle and Regnault, 2001). The phylogenetic position of *C. mobilis* had remained uncertain due to a lack of molecular data, but recently a putative specific relative sharing important morphological and ultrastructural features was isolated from gecko feces and described as *Iotanema spirale* (Yubuki et al., 2017). Phylogenetic analyses based on 18S rRNA gene sequences placed *I. spirale* into Fornicata, specifically into the informal CL3 clade previously established for two free-living marine flagellates *Hicanonectes teleskopos* and a formally undescribed isolate, PCS (Kolisko et al., 2010). Within the CL3 group, *I. spirale*, and presumably also *Caviomonas* spp., are specifically related to the PCS strain (Yubuki et al., 2017). Since *Caviomonas*, *Iotanema*, and PCS are similar in gross morphology, the latter is considered a member of Caviomonadidae (Yubuki et al., 2017). No mitochondrion or MRO candidate was discerned in *I. spirale* by TEM (Yubuki et al., 2017), which contrasts with the presence of a readily observable hydrogenosome-like organelle in *H. teleskopos* (Park et al., 2009); no TEM data was available for PCS. Thus, caviomonads are an insufficiently studied metamonad group that is a good candidate for an independently evolved completely amitochondriate eukaryote lineage.

Besides TEM, the presence/absence and specific features of MROs can be illuminated by genomic or transcriptomic data. For example, the extremely divergent lineage of parasites of marine invertebrates, Microcytida (a subgroup of Rhizaria), is predicted to contain a mitosome based on an analysis of a transcriptome assembly of one of its representatives (Burki et al., 2013), though the organelle has not yet been identified at the cytological level. Some of us previously reported a transcriptome assembly from *I. spirale*, yet this was not analyzed to evaluate the status of the mitochondrion in this organism. Instead, we focused on another interesting aspect of the organism, specifically the use of an unprecedented variant of the nuclear genetic code with the UAG codon reassigned from terminating translation to encode glutamine while keeping UAA as a termination codon (Pánek et al., 2017). This and the simultaneously discovered similar genetic code variant found in an uncultivated member of Rhizaria became the first reported cases of nuclear genetic codes with separate meaning of the two UAR codons, contrasting with >10 previously documented independently evolved variant genetic codes with both UAR codons reassigned to encode an amino acid (Pánek et al., 2017). Hence, the translation system of *I. spirale* is predicted to exhibit unique features that enable it to discriminate between UAG and UAA, but further details as well as the evolutionary origin of the *I. spirale* genetic code are yet to be established. These two unusual features – the potential absence of a mitochondrion and the non-standard genetic code – make caviomonads an attractive subject for further research.

Hence, we set out to further illuminate the biology of caviomonads by exploiting the available transcriptomic resource for *I. spirale* and by obtaining novel data for its free-living relative(s), the marine flagellate PCS. Our results led us to formally describe a new caviomonad genus and species, *Euthynema mutabile*, to clarify the phylogenetic position of caviomonads, to pinpoint the genetic code alteration in this group, and to illuminate the function and evolution of MROs in the caviomonad lineage.

## Materials and Methods

### Culturing and Light Microscopy

The original strain PCS (Kolisko et al., 2010) was maintained in the laboratory in a polyxenic culture with unidentified bacteria in 15 ml tubes with 12 ml of 3:1 (vol:vol) ratio of ATCC (American Type Culture Collection) Seawater Cereal Grass Media #1525^1^ and TYSGM medium (Diamond, 1982) without mucin. The tubes were kept at room temperature (∼23°C) and subcultured three times a week. Clonal cultures, including PCSc10 (see below), were established from individual cells picked from the original culture with a fine glass pipette and kept under the same conditions. Light-microscopic observations were performed using an Olympus BX51 Microscope equipped with an Olympus DP71 camera. Living cells were observed using differential interference contrast, cell size was measured in 20 cells. The measurements were taken from images with the aid of tpsDig2 freeware by F. J. Rohlf^2^. Protargol preparations were prepared by following Nie’s (1950) protocol. A mixture of 1 μl of pelleted culture (centrifugation at 1,100 *g* for 7 min) and 1 μl of egg white diluted 1:4 with the cultivation medium was spread on cover slips and immediately fixed in Bouin-Hollande’s fluid [0.175 M picric acid, 0.138 M copper(II) acetate, 4% formaldehyde, and 5% acetic acid in water] for 6 h, washed with 70% ethanol, and stained with 1% protargol (Bayer, I. G. Farbenindustrie, Frankfurt am Main, Germany; defunct since 1952). Protargol-stained cells were observed with bright-field microscopy.

### Electron Microscopy

For scanning electron microscopy (SEM), 100 μl of a well-grown culture was fixed for 1 h on ice on poly-L-lysine coated coverslips by addition of an equal volume of fixative (a mixture of 1.5% glutaraldehyde and 1% osmium tetroxide). The coverslips were rinsed with distilled water and dehydrated with a graded ethanol series, transferred to acetone and critical point-dried with CO_2_ using a Bal-Tec CPD 030 (Bal-Tec, Witten, Germany). The coverslips were mounted on stubs and coated with gold, using a Bal-Tec SCD 050 sputter coater (Bal-Tec). The cells were observed with a JEOL 6380 LV scanning electron microscope (JEOL Ltd., Tokyo, Japan). For ultra-thin sections, 900 μl of a well-growing culture was prefixed by the addition of 100 μl of 25% glutaraldehyde and pelleted by centrifugation (1,200 *g* for 5 min at 4°C). The supernatant was replaced with 1 mL of 2.5% glutaraldehyde in 0.2 M sodium cacodylate buffer (SCB) and fixed for 1 h on ice. After washing with SCB the sample was postfixed for 1 h on ice by the addition of 1% osmium tetroxide in distilled water and then rinsed with distilled water. Fixed cells were pelleted by centrifugation (1,200 *g* for 5 min) and dehydrated with a graded ethanol series, transferred to acetone and embedded in resin (Epon 812). Ultra-thin sections were cut on a Reichert-Jung Ultracut-E microtome (Reichert-Jung, Vienna, Austria) with a diamond knife, stained with uranyl acetate and lead citrate (Reynolds, 1963) and observed using a JEM 1011 transmission electron microscope (JEOL Ltd., Tokyo, Japan).

### Transcriptome Sequencing, Processing, and Evaluation

Total RNA from cell pellets of the original culture PCS and the clonal subculture PCSc10 was extracted from 400 ml of well-grown cultures using Tri Reagent (Sigma-Aldrich) and chloroform, further purified using the RNeasy mini kit (QIAGEN), and treated with DNase (QIAGEN). Transcriptome sequencing was performed by Macrogen (Seoul, South Korea) with the Illumina platform and paired-end sequencing strategy. For the RNA sample from the original PCS culture, the sequencing library was prepared using TruSeq RNA Sample Prep Kit v2 and 38,903,290 reads (read length: 101 bp) were obtained. From the PCSc10 clonal culture, the sequencing library was prepared using TruSeq stranded mRNA kit and 44,372,620 reads (read length: 151 bp) were obtained. *De novo* transcriptome assemblies were performed for both datasets using Trinity v2.11.0 with –trimmomatic flag for trimming of the Illumina adapter and low-quality bases (Grabherr et al., 2011). To obtain a transcriptome assembly for the second CL3 clade representative organism contaminating the original PCS culture (“PCS-ghost”; see Section “Results and Discussion”), the sequence data from the PCS culture were subtracted with the PCSc10 assembly. Bowtie2 v2.3.5.1 (Langmead and Salzberg, 2012) with default settings was used to map trimmed Illumina reads from the PCS (“ghost-contaminated”) culture onto the assembly from the PCSc10 (“ghost-free”) culture. The generated sequence alignment (SAM) files were manipulated with SAMtools v1.10 (Li et al., 2009). The unmapped reads (7,425,970 in total) were retained and separately assembled with Trinity (without –trimmomatic option). The representativeness (“completeness”) of the assemblies was evaluated with BUSCO v5.0.0 (Simão et al., 2015) with default settings using the eukaryota_odb10 dataset. Additionally, each assembly was scored for the number of identified orthologs of genes included in the core dataset of the PhyloFisher package (Tice et al., 2021). An ortholog was assigned only when obeying the strict default PhyloFisher standards. The resulting range is between 0 and 1, where 0 means no orthologs from the PhyloFisher database were found in the particular assembly, and 1 means that all orthologs were identified.

### Phylotranscriptomic Analyses and Genetic Code Evaluation

TransDecoder v5.5.0 (Haas and Papanicolaou, 2017) was used to predict protein sequences encoded by transcriptome assemblies from PCSc10 (below described as *Euthynema mutabile*), PCS-ghost, *Iotanema spirale*, *Dysnectes brevis*, *Chilomastix cuspidata*, *Chilomastix caulleryi*, *Retortamonas dobelli*, and *Barthelona* sp. PAP020 (all sources of sequence data are listed in Supplementary Table 1). Phylogenetic analyses were performed using two runs of PhyloFisher package v1.0.0, following the protocol recommended by the authors (Tice et al., 2021). The program fisher.py was used to collect all candidate orthologous sequences corresponding to the 240 conserved eukaryotic genes constituting the core PhyloFisher database. All organisms and genes from the database were kept and the programs working_dataset_constructor.py and sgt_constructor.py were then used to prepare single gene trees. In the next step forest.py was used to convert the resulting trees to a format suitable for ParaSorter. After manual inspection and curation of all trees, all newly selected orthologs were added to the main PhyloFisher database with the use of apply_to_db.py. Possible stop-to-sense and sense-to-sense codon reassignments in the taxa examined were assessed with the aid of the program genetic_code_examiner.py from the PhyloFisher package, with –prepare_alignments flag on in the first run and all available sequences from Metamonada in PhyloFisher core database as –queries for genetic code examination. For the final phylogenetic reconstruction a subset of taxa was selected with select_taxa.py, the dataset prepared with prep_final_dataset.py, and the matrix generated with matrix_constructor.py, selecting 180 genes that were represented in at least 75% of the taxa included in the analysis. Tree inference on the matrix was carried out with the maximum likelihood (ML) method using IQ-TREE v2.0.3 (Nguyen et al., 2015). A guide tree was obtained by an analysis under the LG + F + G model and the final phylogenomic tree was then computed using the LG + F + C20 + G with posterior mean site frequency (PMSF) approximation to a site-heterogeneous mixture model (Wang et al., 2018) with 100 non-parametric bootstrap pseudoreplicates.

### Single-Gene Phylogenetic Analyses

For phylogenetic analysis of the SSU rRNA gene, reference sequences from all main Fornicata lineages and a set of other eukaryotic taxa as an outgroup were retrieved from GenBank and combined with SSU rRNA sequences of *E. mutabile* (PCSc10) and PCS-ghost retrieved from the respective transcriptome assemblies. The sequences were aligned using the on-line tool MAFFT version 7^3^ (Katoh et al., 2019) with the G-INS-i algorithm at default settings. The alignment was manually edited in BioEdit 7.0.9.0 (Hall, 1999). Unreliably aligned positions were manually trimmed to yield the final alignment of 1,374 positions, which was subjected to phylogenetic inference by the ML method in RAxML 8.0.0 (Stamatakis, 2014) under the GTRGAMMAI model. Bootstrap support values were calculated in RAxML from 1,000 pseudoreplicates. Phylogenetic analyses of α- and β-tubulin, EF1α, and HSP90 were carried out using the corresponding set of amino acid sequences adopted from the study by Takishita et al. (2012), updated with the addition of orthologs from CL3 clade organisms and other relevant taxa. The sets of sequences for the phylogenetic analysis of PFO and PFL proteins were assembled by combining the respective sequences obtained from the transcriptome assemblies of CL3 clade organisms with sequences selected by blastp searches against the EukProt database (Richter et al., 2020), transcriptome assemblies of individual metamonads, and the non-redundant NCBI protein sequence database. The queried EukProt database was compiled into a single blastable database by replacing the uninformative, inconsistent and potentially redundant sequence headers of the sequences by unified species-diagnostic titles and then merging all the files into one. For the phylogenetic analysis of HydA proteins the set of sequences analyzed by Leger et al. (2017) was adopted and expanded by adding homologs from CL3 clade members. Multiple alignments were obtained using MAFFT (default settings) and trimmed using trimAl v. 1.2rev57 (gappyout setting; Capella-Gutiérrez et al., 2009). Trees were inferred using IQ-TREE v2.1.2, with the substitution model selected by the program (the model specific for the given analysis is indicated in the figure legend of the respective phylogenetic tree). Branch support was estimated with SH-aLRT and ultrafast bootstrapping (10,000 pseudoreplicates) as implemented in IQ-TREE. Trees were visualized and adjusted in iTOL^4^ (Letunic and Bork, 2021) or FigTree v1.4.4,^5^ exported as svg files and graphically edited and annotated for clarity in Inkscape.^6^

### Searching for Candidate Mitochondrion-Related Organelle-Localized Metabolism Proteins

Proteins potentially localized to or functionally associated with the MROs in the three focal species were searched with tblastn (Altschul et al., 1997), targeting the respective transcriptome assemblies, or HMMER 3.0 (Eddy, 2011), querying the sets of protein sequences inferred from the respective transcriptomes. In the case of *I. spirale* we searched not only the previously released transcriptome assembly (GenBank GFCE00000000.1) that was filtered for bacterial contamination (Pánek et al., 2017), but also the original unfiltered assembly, where we identified additional authentic *I. spirale* sequences that had been mistakenly recognized as bacterial. Homologs of proteins of interest were also searched in transcriptome assemblies or inferred sets of protein sequences from other fornicates (sources of data provided in Supplementary Table 1). As queries for tblastn searches we used reference sequences of interest from common model organisms and from previously analyzed metamonads (Leger et al., 2017; Tachezy and Šmíd, 2019; Stairs et al., 2021). For HMMER searches, profile hidden Markov models (HMMs) were built from seed alignments of the respective Pfam families^7^ (Mistry et al., 2021) or from custom alignments comprised of previously identified metamonad proteins (as in the case of the Tim17 protein; Pyrihová et al., 2018). Significant hits (tblastn: *e*-value ≥ 1e-5; HMMER: e-value above the inclusion threshold) were evaluated by blastx or blastp against the NCBI non-redundant protein sequence database or by phylogenetic analyses to confirm the homolog identification and to distinguish bacterial contaminants from authentic sequences of the CL3 representatives analyzed. Subcellular localization of the candidate proteins was predicted using TargetP 2.0 (‘‘non-plant’’ setting^8^ Almagro Armenteros et al., 2019), MitoFates (set to ‘‘fungi’’^9^; Fukasawa et al., 2015), and NommPred (Kume et al., 2018).

## Results and Discussion

### The Original PCS Culture Was a Mixture of Two Different Fornicates

The culture PCS was obtained from littoral marine anoxic sediments in Prince Cove, Marstons Mills, MA, United States (Kolisko et al., 2010). According to our initial light-microscopy observations, it appeared monoeukaryotic and included a flagellate with elongated cells possessing a single anterior flagellum and lacking a conspicuous feeding groove, as reported by Kolisko et al. (2010; see their Figures 1O,P). The culture was used for RNA isolation and transcriptome sequencing using Illumina HiSeq, which yielded 30,528 contigs after RNAseq read assembly. Strikingly, analyses of the transcriptome assembly suggested that two different eukaryotes had contributed to it (disregarding a minor fungal contamination). This was obvious by the presence of contigs representing two different versions of the 18S rRNA gene as well as quasi-duplications of various protein-coding genes. One of the 18S rRNA sequence was nearly identical to the previously reported partial sequence assigned to the PCS strain (GenBank GU827590.1; Kolisko et al., 2010), whereas the other was more similar to the 18S rRNA previously reported from the “*Carpediemonas*-like” fornicate *H. teleskopos* (Park et al., 2009). We concluded that the original PCS culture in fact contained two different, presumably morphologically similar and phylogenetically close relatives, one corresponding to the previously reported PCS genotype and the other representing a novel member of the CL3 clade, hereafter referred to as PCS-ghost.

**FIGURE 1:**
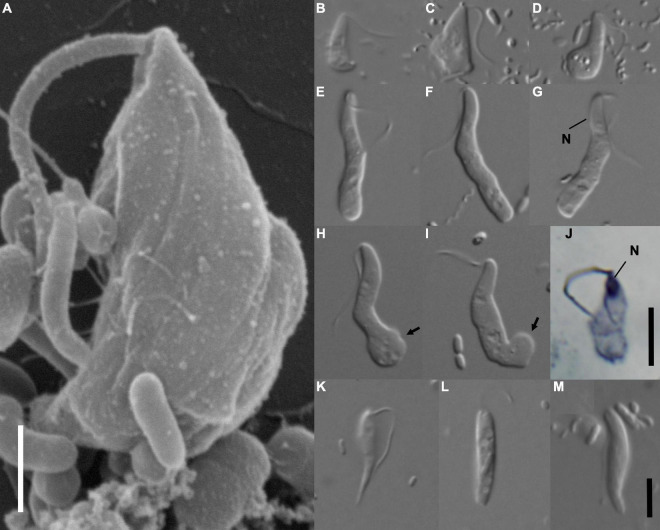
Morphology of *Euthynema mutabile* sp. nov., strain PCSc10 **(A–J)**, and cells in the original PCS culture potentially corresponding to the PCS-ghost genotype **(K–M)**. **(A)** Scanning electron micrograph, lateral view showing body shape, a single flagellum and absence of a feeding groove, scale bar 5 μm. **(B–I)** Light micrographs of living cells *E. mutabile* sp. nov., strain PCSc10, differential interference contrast. Adherent form **(B,C)**. Swimming form **(E–I)**. **(J)** Light micrograph of a protargol-stained cell showing the nucleus, bright field, scale bar 5 μm. **(K–M)** Morphotypes potentially representing PCS-ghost; note the pointed cell posterior **(K)**. N, nucleus; arrow, adhesive pad. Scale bar in **(M)** represents 5 μm and holds for **(B–I,K–M)**.

**FIGURE 2:**
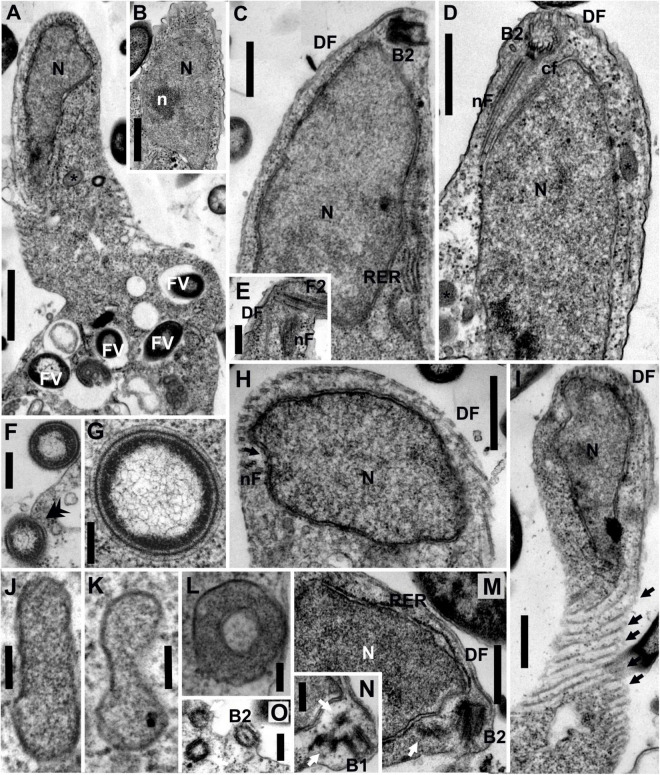
Transmission electron micrographs of *Euthynema mutabile* sp. nov., strain PCSc10, ultra-thin sections. **(A)** Longitudinal section through the cell, showing the position of the nucleus and the presence of food vacuoles. **(B)** Oblique section of the nucleus showing the presence of a nucleolus. **(C)** Longitudinal section of the anterior part of the cell, lateral view, showing the nucleus and the position of basal body 2 (B2). Note rough endoplasmic reticulum (RER). **(D)** Longitudinal section of the anterior part of the cell, ventral view, showing the nucleus and the position of basal body 2 (B2) and microtubular fibers. **(E)** Detail of the anterior part of the cell showing the flagellum, B2 and microtubular fibers. **(F)** Phagocytosis of bacteria (arrow). **(G)** Putative bacterial endosymbiont. **(H)** Oblique section of the anterior part of the cell. **(I)** Grazing section of the surface of the cell showing the microtubules of the dorsal fan (arrows). **(J–L)** High magnification of putative mitochondrion-related organelles (MROs). **(M,N)** Oblique sections of the anterior part of the cell showing the position of basal bodies (arrows show lateral BBs). **(O)** Oblique section of the anterior part of the cell showing BBs. DF, dorsal fan of microtubules; FV, food vacuole; cF, non-microtubular connecting fiber; N, nucleus; n, nucleolus; nF, nuclear fiber; RER, rough endoplasmic reticulum; asterisk (*), putative mitochondrion-related organelle; black arrow, individual microtubules of DF; double arrowhead, phagocytosis; white arrow, lateral basal bodies. Scale bars: 1 μm for **(A)**; 500 nm for **(B-D,H,I)**; 250 nm for **(E,F,M,N,O)**; 200 nm for **(G)**; 100 nm for **(J-L)**.

To obtain a truly monoeukaryotic culture, we established 14 clonal cultures derived from individually picked cells. The purity of each subculture was tested by PCR amplification and partial sequencing of the 18S rRNA gene, which suggested that only the organism corresponding to the originally reported PCS genotype was present in all of them. The clonal culture PCSc10 was selected for further investigation. Its monoeukaryotic status was confirmed by a new round of transcriptome sequencing, which provided 28,567 contigs without the apparent presence of the PCS-ghost genotype (i.e., the respective 18S rRNA gene or the extra copies of the protein-coding genes inspected). All the morphological (Figures 1A–J) and ultrastructural (Figure 2) investigations reported below, and the description of the new genus and species *E. mutabile*, are based on the clonal subculture PCSc10, and for all subsequent genetic analyses concerning this organism, the transcriptome assembly from PCSc10 has been used. We reasoned that the PCS-ghost component of the transcriptome data obtained from the original mixed PCS culture may provide a useful resource for exploring the biology and evolution of the CL3 clade, especially considering the fact that the original culture of *H. teleskopos* was lost before transcriptome or genome data could be obtained from it. To extract the PCS-ghost component from the mixed data, we filtered the reads from the original RNAseq run by removing those that could be mapped to the transcriptome assembly obtained from the monoeukaryotic clonal PCSc10 culture. The remaining reads were reassembled into a set of 16,810 contigs.

The complete 18S rRNA genes of PCSc10 and PCS-ghost were retrieved from their respective transcriptomes. The PCSc10 18S rRNA gene sequence was 99% identical to the previously reported incomplete PCS sequence (Kolisko et al., 2010). Phylogenetic analysis with the updated or newly obtained sequences (Supplementary Figure 1) confirmed the previous result (Yubuki et al., 2017) that *E. mutabile* (PCSc10) is most closely related to an unidentified, presumably free-living eukaryote represented by the sequence clone D4P08A09 obtained from oxygen-depleted intertidal marine sediment in Greenland (GenBank accession number EF100347.1, Stoeck et al., 2007). These two OTUs form a clade with *I. spirale*, and the three together represent the caviomonad clade. PCS-ghost and *H. teleskopos* formed a clade that is a sister lineage to Caviomonadidae. All these relationships received full bootstrap support, but the position of the whole Caviomonadidae-*Hicanonectes*-PCS-ghost clade, i.e., the fornicate lineage CL3 as originally defined by Kolisko et al. (2010), was unresolved (Supplementary Figure 1).

The organism corresponding to transcriptomically recorded PCS-ghost was not obtained in a pure culture and its morphology remains undefined. Nevertheless, we observed a specific morphotype in the original PCS culture absent from the clonal culture PCSc10. These cells had a pointed posterior end and were able to glide on their flagellum (Figures 1K–M). This morphotype might represent PCS-ghost. On the other hand, we cannot rule out the possibility that the disappearance of this morphotype from the clonal PCSc10 isolate was caused, for example, by changes in the composition of the bacterial community during subculturing. At any rate, no cells morphologically reminiscent of *H. teleskopos*, i.e., a fornicate potentially related to PCS-ghost (but see below) and characterized by cells with two flagella and a conspicuous ventral groove (see Park et al., 2009), were observed at any stage of PCS culturing, which is also consistent with the observations of the original PCS culture by Kolisko et al. (2010).

### Morphology and Ultrastructure of a Novel Free-Living Caviomonad, *Euthynema mutabile*, gen. et sp. nov., Clonal Culture PCSc10

The living cells of *E. mutabile* had a simple general morphology. The cells were elongated, had a single anterior flagellum without any modifications (i.e., no flagellar vanes), and lacked an obvious feeding groove (Figure 1A). The nucleus was located in the anterior part of the cell close to the flagellar insertion (Figures 1G,J). The cells fed on bacteria by phagocytosis, which occurred in the posterior part of the cell body. Two distinct cell-types were present in the culture, a swimming form (Figures 1E–I) and an adherent form (Figures 1B–D). The swimming form was elongated, on average 16.2 μm long (S.D. ± 1.5; range 13.6–18.8 μm) and 3 μm wide (S.D. ± 0.4; range 2.0–3.8 μm), with an average length/width ratio of 5.6 (*n* = 20, live cells). The anterior third of the cell was finger-like and had a stable shape, while the rest of the cell was pleomorphic and sack-like. The length of the flagellum was two thirds of the cell body length. It was directed posterolaterally and beat with a sinusoid pattern. The cell swam with a slow rotation (approximately one rotation per second). The swimming form occasionally adhered to the slide, using an adhesive pad formed from the posterior end of the cell, while the anterior part of the cell remained free and slowly rotated (Figures 1H,I). The adhesive form was pear-shaped, on average 8.2 μm long (S.D. ± 0.8; range 6.7–9.8 μm) and 3.5 μm wide (S.D. ± 0.6; range 2.7–4.5 μm), with an average length/width ratio of 2.4 (*n* = 20, live cells). The flagellum was as long as the cell body (Figures 1B–D). The cells never produced pseudopodia. The swimming form predominated in cultures that were passed every 3 days, while the adherent form predominated in cultures that were passed once a week.

The nucleus of *E. mutabile* was irregular in shape and typically contained a single nucleolus (Figures 2A,B). It was located anteriorly, close to the basal bodies, and was surrounded by cisternae of the rough endoplasmic reticulum (RER). Cisternae of RER were also located beneath the cell membrane on the ventral side of the cell (Figure 2C). A large number of food vacuoles with partially digested bacteria were present in the sack-like posterior part of the cell. Phagocytosis of bacteria without the involvement of the microtubular cytoskeleton was observed in the posterior part of the cell (Figures 2A,F). Several acristate mitochondrion-related organelles (MROs) were observed in the cell (Figures 2J–L). They were scattered throughout the cytoplasm and were shaped as oval biconcave discs, 250–600 nm in length and 80–100 nm in width (in cross section; *n* = 10). The bounding membrane appeared to be doubled; however, the quality of fixation was suboptimal. Prokaryotic cells lying free in the *E. mutabile* cytoplasm, which may be endosymbionts, were observed (Figure 2G). A discrete Golgi dictyosome was not observed.

The flagellar apparatus of *E. mutabile* was simple and very similar to that of *I. spirale*. A single naked flagellum arose from the basal body B2. Only two microtubular elements were present, a nuclear fiber (nF) and a dorsal fan (DF) (Figures 2D,E). The nF was the only microtubular root in the mastigont of *E. mutabile*. Identical to *I. spirale*, it consisted of three microtubules that originated in the vicinity of B2, extended posteriorly through a gutter-like concavity of the nucleus and lay just beneath the cell membrane (Figures 2D,E,H). The nF was closely associated with a non-microtubular connecting fiber (cF), which interconnected nF and B2 (Figure 2D). The multilayered fiber described in *I. spirale* was not observed in *E. mutabile*. The dorsal side of the cell was supported by a fan of radiating microtubules (dorsal fan; DF) that originated close to B2 and extended posteriorly immediately beneath the cell membrane (Figures 2C,D,H,I). In addition to B2, which bears the flagellum, three barren basal bodies were observed in several cells, basal body B1 and two lateral basal bodies BL (Figures 2M–O).

The ultrastructural features observed in the mastigont of *E. mutabile* correspond well with the placement of the organism in the family Caviomonadidae. The mastigont is highly reduced in comparison with other fornicates, including the putative closest relative of described caviomonads (but see below), *H. teleskopos* (Park et al., 2009), and the organization of the microtubular elements is very similar to *I. spirale* (Yubuki et al., 2017). The morphology of *E. mutabile* and other caviomonads robustly delineate the family Caviomonadidae with the following characters: (1) single flagellum devoid of vanes; (2) elongate cell-shape; (3) absence of a feeding groove; (4) simple flagellar apparatus; and (5) one microtubular root (nuclear fiber) and a dorsal fan of microtubules originating at the B2. The major ultrastructural difference between *E. mutabile* and other caviomonads is the presence of several putative MROs; no such structures are apparent in either *C. mobilis* or *I. spirale* (Brugerolle and Regnault, 2001; Yubuki et al., 2017). Consistent with other fornicates (Kulda et al., 2017), the MROs in *E. mutabile* were double-membrane bounded and acristate. Interestingly, they were shaped like oval biconcave disks, while MROs of other fornicates are typically rounded. Although unusual, a similar MRO morphology has been observed in the unrelated anaerobic heterolobosean, *Sawyeria marylandensis* (Barberà et al., 2010). The putative bacterial endosymbionts observed in the cytoplasm of *E. mutabile* were not associated with the MROs and remotely resembled the prokaryotes observed in the cytoplasm of *H. teleskopos* (Park et al., 2009).

### Pinpointing the Genetic Code Change in Caviomonads

Manual tblastn searches of the *E. mutabile* (i.e., PCSc10) transcriptome assembly with various conserved protein sequences did not indicate the presence of in-frame termination codons in coding sequences, and the UAG codon was found in multiple transcripts in the region corresponding to the presumed coding sequence end defined on the basis of conservation of the respective encoded proteins. In addition, we employed a specific tool for genetic code analysis that is part of the recently developed PhyloFisher package and can detect stop-to-sense and sense-to-sense codon reassignments (Tice et al., 2021); no departures from the standard code were suggested by this tool. Manual identification of termination codons in a sample of 170 *E. mutabile* genes revealed that seven (i.e., 4.12%) of them apparently use UAG to terminate translation. Hence, we conclude that *E. mutabile* does not share the stop-to-Gln reassignment of the UAG codon previously discovered in *I. spirale* (Pánek et al., 2017), although the apparently low usage of UAG in *E. mutabile* may indicate that the process of abandoning UAG as a termination codon may have started early in caviomonad evolution. Just like in *E. mutabile*, there is no evidence for a reassignment of the UAG codon in the transcriptome data from PCS-ghost, consistent with this organism being even more distantly related to *I. spirale* than *E. mutabile*.

It was previously reported that the protein responsible for termination codon recognition, eRF1, bears a specific mutation in *I. spirale* presumably related to the reassignment of the UAG codon (Pánek et al., 2017). Specifically, the highly conserved amino acid motif GTS that is critical for its functioning is mutated in *I. spirale* such that the threonine residue is replaced by a glycine residue, thus disrupting a hydrogen bond between eRF1 and the guanine base in the UAG codon. We identified transcripts encoding the eRF1 protein in both *E. mutabile* and PCS-ghost, and interestingly, they both have preserved the standard GTS motif (Supplementary Figure 2). This observation lends credence to the notion that the mutation in the GTS motif in the *I. spirale* eRF1 is mechanistically linked to the UAG codon being translated as an amino acid rather than recognized as a termination codon. Altogether, our analyses establish that the genetic code change is specific for the *Iotanema* lineage within the CL3 clade. Future molecular characterization of *Caviomonas mobilis* may further illuminate the evolutionary path toward the non-standard code in *I. spirale* and whether the change is possibly associated with the switch to an endobiotic lifestyle in caviomonads.

### Caviomonad Phylogeny Illuminated by Phylotranscriptomic Analysis

While the 18S rRNA gene alone does not resolve the exact position of the CL3 clade in fornicates (Supplementary Figure 1), previous phylogenetic analyses of concatenated sequences of six protein markers (alone or in combination with 18S rRNA) suggested that CL3 is specifically related to the fornicate lineage CL2 (Takishita et al., 2012), represented by *Aduncisulcus paluster* (later formally described by Yubuki et al., 2016). The lack of transcriptome data from CL3 representatives prevented testing this potential higher-order grouping within Fornicata in the previous nearly comprehensive multigene analysis of fornicate phylogeny (Leger et al., 2017). To address the phylogenetic position of caviomonads and the whole CL3 clade by a phylogenomic (or, more precisely, phylotranscriptomic) approach, we employed the PhyloFisher package to expand the existing core 240-gene dataset defined by the program, which already includes many fornicate and metamonad representatives (Tice et al., 2021), by adding orthologs from additional taxa so that all main Fornicata lineages were represented in the alignments. We also included sequences from *Barthelona* sp. PA020, a metamonad lineage most closely related to fornicates (Yazaki et al., 2020).

During the process of parsing individual gene trees to ensure that only orthologs were included in the final phylogenomic analyses, there were 11 cases in which two homologs were present in the filtered PCS-ghost transcriptome. In each case, one of the homologs was nearly identical at the amino acid sequence level to a corresponding *E. mutabile* protein and the other was more distantly related within the CL3 clade. This indicates the likely presence of *E. mutabile* allelic variants that were expressed in the original mixed PCS culture but not in the clonal culture and thus, not removed when subtracting the clonal RNAseq data from that of the original PCS culture. Therefore, when assigning genes to PCS-ghost, we discarded the homolog that was nearly identical to the homologous *E. mutabile* protein and assigned the other homolog to PCS-ghost. The final concatenated alignment consisted of 49,027 amino acid positions from 180 genes and 27 taxa (including representatives of other metamonad lineages and a selection of other eukaryotes), and was used to infer a tree by the maximum likelihood (ML) method and a complex substitution model approximating a full empirical profile mixture model (LG + F + C20 + G + PMSF).

**FIGURE 3:**
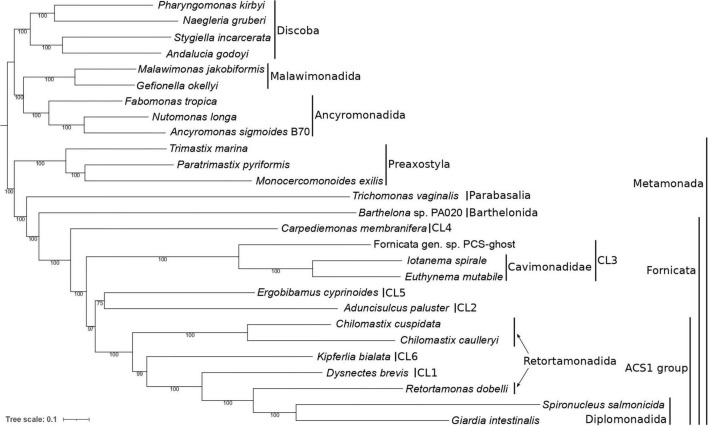
Phylogenetic position of caviomonads inferred from a dataset of 180 conserved proteins (49,027 amino acid positions, 27 taxa). The tree was inferred with the maximum likelihood method using IQ-TREE v2.0.3 and the LG + F + C20 + G + PMSF substitution model. The root is arbitrarily placed between Metamonada and other eukaryotic groups included in the analysis. Numbers at branches indicate support values calculated from 100 non-parametric bootstrap pseudoreplicates. Major subgroups of Metamonada and Fornicata are delineated by vertical bars. CL1 to CL6 correspond to clades of *Carpediemonas*-like organisms as defined by Kolisko et al. (2010). The clade denoted “ACS1” is characterized by the presence of the ACS1 form of acetyl-CoA synthase (see main text). Note that Retortamonadida (the genera *Chilomastix* and *Retortamonas*) are polyphyletic. PCS-ghost corresponds to the uncharacterized organism in the original PCS culture (see main text).

The resulting tree confirmed the monophyly of Fornicata (Figure 3), with the internal topology of the group in general agreement with previous multigene analyses (Leger et al., 2017; Stairs et al., 2021). *Carpediemonas membranifera* (CL4) branched sister to all other fornicates, followed by a fully supported CL3 clade comprising PCS-ghost as sister to the described caviomonads. A moderately supported grouping (bootstrap value of 75%) of *Aduncisulcus paluster* (CL2) and *Ergobibamus cyprinoides* (CL5) was sister with a high bootstrap value (97%) to a fully supported clade where *Retortamonas dobelli*, *D. brevis*, *Kipferlia bialata*, and *Chilomastix* spp. appear as specific, successively more distant relatives of diplomonads. We denote the grouping comprised of diplomonads and their relatives up to *Chilomastix* spp. as the ACS1 group, since these fornicates are characterized by the shared possession of the ACS1 form of acetyl-CoA synthase that presumably replaced the ACS2 form ancestral for fornicates (Leger et al., 2017; Füssy et al., 2021). Interestingly, previous analyses recovered *A. paluster* (CL2) and *E. cyprinoides* (CL5) as independent lineages, with *A. paluster* branching sister to the ACS1 group (bootstrap value of 88%) and *E. cyprinoides* sister to both these taxa combined (Leger et al., 2017). The topological differences between the two analyses and the relatively low statistical support for the placement of *A. paluster* and *E. cyprinoides* indicate some level of a conflicting phylogenetic signal in this area of the fornicate phylogeny that calls for further analyses, ideally with greater taxon representation from the CL2 and CL5 clades.

Furthermore, our phylogenomic analysis is in an obvious conflict with the results of six- and seven-gene phylogenies reported by Takishita et al. (2012) that suggested a different position of the CL3 clade, which was represented only by *H. teleskopos*, uniting it with full support with the CL2 clade. However, we strongly question (see Supplementary Text 1) that the published genes ascribed to *H. teleskopos* actually belong to one taxon because single-gene analyses reveal conflicting phylogenic affiliations. In particular, serious doubts concern the origin of the putative *H. teleskopos* 18S rRNA gene sequence, the only marker suggesting a close relationship of this organism to PCS-ghost or caviomonads (Supplementary Figures 3–6). Given these ambiguities, the inclusion of *H. teleskopos* into either CL3 or CL2 should be considered provisional and the conclusions by Takishita et al. (2012) on the specific relationship of the CL3 and CL2 lineages were perhaps premature. The taxonomic assignment of *Hicanonectes* into a particular fornicate lineage awaits its re-isolation for inclusion into rigorous phylogenomic analyses. Thus, the CL3 lineage circumscribed here comprises PCS-ghost, *Euthynema*, *Iotanema* and (based on morphological and ultrastructural evidence only) *Caviomonas*. Future cytological investigations PCS-ghost are critical to test the possibility for its inclusion into the family Caviomonadidae. Additional work, ideally incorporating increased fornicate diversity, including verified sequence data from *H. teleskopos*, is also needed to settle the unresolved position of *A. paluster* and *E. cyprinoides* in the phylotranscriptomic analyses.

### *In silico* Evidence for a Mitochondrion-Related Organelle in *Iotanema spirale*

The availability of transcriptome assemblies established for caviomonads in this and a previous study (Pánek et al., 2017), together with the serendipitously obtained (meta)transcriptome data for PCS-ghost, provide an opportunity to get the first insights into the features of the MRO in the CL3 lineage. To evaluate the degree to which the transcriptome assemblies from *I. spirale*, *E. mutabile*, and PCS-ghost represent the full gene repertoire of the respective species, we analyzed them with BUSCO. The BUSCO scores of the focal species are generally low, from 34.5% (*I. spirale*) to 42.0% (PCS-ghost) of the BUSCO reference genes represented (as complete or fragmented) in the transcriptome assemblies. However, these values fit into the range exhibited by transcriptome assemblies reported from other metamonads, with some of them (*K. bialata*, *C. caulleryi*, *E. cyprinoides*, and *R. dobelli*) having even lower scores than the assembly from *I. spirale* (Supplementary Figure 7A). While it is certain that the transcriptome assemblies miss many genes that are in fact present in the respective organisms, the low BUSCO values probably reflect a generally reduced gene complement in fornicate genomes and the divergent nature of their gene sequences. This is also apparent when considering the BUSCO scores of complete genome assemblies of diplomonads (Supplementary Figure 7A). As an alternative measure we plotted the representation in transcriptome or genome assemblies of orthologs of the 240 genes constituting the core phylogenomic dataset of the PhyloFisher package. The caviomonad and PCS-ghost transcriptome assemblies scored better than some of the previously reported fornicate transcriptome assemblies and even better than the diplomonad genome sequences (Supplementary Figure 7B). Hence, these analyses indicated that the caviomonad and PCS-ghost transcriptome assemblies are as good for comparative genomic reconstructions of particular functional systems (including MROs) as those used for other metamonads in previous studies (Leger et al., 2017; Yazaki et al., 2020; Füssy et al., 2021).

We then searched the transcriptome assemblies of the three CL3 clade representatives for homologs of MRO-associated proteins, including those whose distribution in fornicates was systematically analyzed by Leger et al. (2017). For N-terminally complete sequences, their possible MRO localization was evaluated by three prediction programs, including one specifically developed to improve the prediction accuracy for organisms with MROs (NommPred, Kume et al., 2018). However, when it comes to recognizing MRO-localized proteins, *in silico* methods are generally less reliable compared to prediction of proteins that localize to “standard” aerobic mitochondria owing to the often poorly defined or divergent MRO-localization signals (Rada et al., 2015; Dolezal et al., 2019). Thus, those proteins predicted to be mitochondrion- or MRO-targeted by at least one of these tools are considered here as strong candidates for MRO-localized proteins (Supplementary Tables 2, 3). However, a negative result for MRO targeting of a protein by all three programs used should not be automatically considered as evidence against its localization in the organelle (see below specific discussion for cases of special interest). Even though the subtraction of *E. mutabile* transcripts from the original PCS transcriptome data was efficient (because of the excess primary data originating from the clonal *E. mutabile* culture), we were extremely cautious when interpreting the gene content within the PCS-ghost transcriptome data because of the possibility that it may contain a minor, cryptic, component derived from *E. mutabile*. Indeed, there were three genes with two versions in the PCS-ghost transcriptome that are part of the analyses to identify MRO-associated proteins. Similar to the construction of the phylotranscriptomic data set (described above), one version was always extremely similar at the amino acid sequence level to and ascribed to *E. mutabile* with the other assigned to PCS-ghost (Supplementary Tables 2, 3). Therefore, the few single-copy genes of undisputed fornicate origin identified in the PCS-assembly that are not present in the *E. mutabile* assembly (HydE to HydG, serine hydroxymethyltransferase, see below) are unlikely to be an *E. mutable* carryover or ‘contamination’ and can be confidently, but cautiously, assigned to PCS-ghost.

**FIGURE 4:**
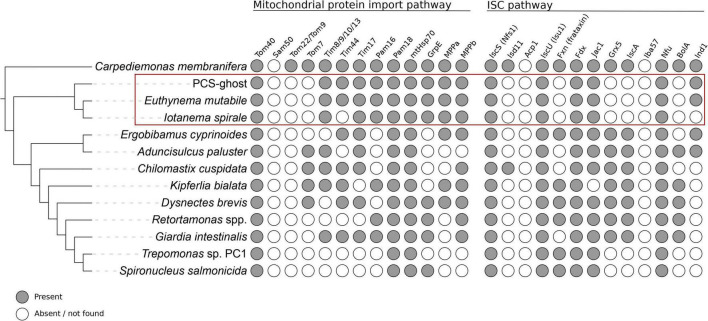
Occurrence of proteins homologous to components of the mitochondrial protein import and ISC pathways in CL3 clade members and other selected metamonads. Data from species outside the CL3 clade come from previous studies (Leger et al., 2017; Yazaki et al., 2020; Füssy et al., 2021; Stairs et al., 2021) and updated by additional homology searches (the data for the update are listed in Supplementary Table 5).

With these caveats in mind, we first looked for proteins that constitute the basic molecular machinery responsible for the organelle’s biogenesis (Dolezal et al., 2019). Crucially, both caviomonads and PCS-ghost possess homologs of components of the apparatus mediating mitochondrial protein import, including the β-barrel protein Tom40 that forms the protein-conduction channel in the mitochondrial and MRO outer membrane, a homolog of the “small Tims” (Tim8/9/10/13) that function in the intermembrane space (IMS) as chaperons for the delivery of hydrophobic protein substrates to the inner mitochondrial membrane, an ortholog of the Tim17 protein thought to constitute a protein-translocating pore in the inner mitosomal membrane of *Giardia intestinalis* (Pyrihová et al., 2018), the components of the PAM complex that functions as a “motor” to pull the imported protein substrates into the mitochondrial (or MRO) matrix, i.e., Pam16, Pam18, mtHsp70, and GrpE, and both subunits of mitochondrial processing peptidase (MPP) that cleaves the N-terminal targeting signals from proteins imported into mitochondria and MROs (Figure 4 and Supplementary Table 2). The only difference among the three organisms investigated concerning the MRO protein import machinery is in the apparent absence of Tim44 from *I. spirale*, shared with some other fornicates, including *S. salmonicida* with fully sequenced genome (Figure 4). No candidate Tim44 homolog could be identified in these organisms even when using a custom profile HMM built from Tim44 sequences identified in other fornicates, suggesting that this component is not essential. Like other fornicates thus far analyzed, none of the CL3 clade representatives possess homologs of the IMS-localized disulfide relay system recently identified in *Anaeramoeba* spp., a newly recognized major metamonad lineage (Stairs et al., 2021).

The so-called ISC pathway for the assembly of iron-sulfur (Fe-S) clusters is the most widespread mitochondrial functionality retained, even in most highly reduced MRO forms (Tachezy and Šmíd, 2019; Braymer et al., 2021), although exceptions do exist (Santos et al., 2018; Leger et al., 2019; Lill and Freibert, 2020). The transcriptome assemblies of the three CL3 representatives were queried and indeed, all of them possessed homologs of essential components of the ISC pathway, including cysteine desulfurase (IscS), the scaffold protein for Fe-S cluster assembly (IscU), the ISC pathway-specific DnaJ domain-containing regulator of mtHsp70 (Jac1), and [2Fe-2S]ferredoxin (Fdx, also called Yah1), which provides reducing power to the ISC pathway in mitochondria and MROs (Figure 4 and Supplementary Table 2). Nevertheless, the set of ISC pathway components detected in the CL3 clade members is noticeably reduced when compared to the complements found in many other fornicates, including *Giardia* (Figure 4 and Supplementary Table 4). This specifically concerns the various “late-acting” protein factors that mediate trafficking and targeting of Fe-S clusters to client proteins (Tachezy and Šmíd, 2019; Braymer et al., 2021), including IscA, Grx5, and BolA; the only such factor present, conserved in all three CL3 clade species, is Nfu (Figure 4 and Supplementary Table 2). Some of the missing factors are implicated in the synthesis of the [4Fe-4S] type of clusters, which is of concern given the requirement of these clusters for the functioning of some of the enzymes (hydrogenase and pyruvate:ferredoxin oxidoreductase) whose presence in MROs of the CL3 clade representatives is considered below. We note, however, that the diplomonad *S. salmonicida*, experimentally proven to have these enzymes functioning in its MRO (Jerlström-Hultqvist et al., 2013), lacks exactly the same set of Fe-S cluster carriers while keeping Nfu, as in the CL3 clade taxa (Figure 4 and Supplementary Table 4; see also Braymer et al., 2021). It is therefore probable that an alternative, hitherto uncharacterized, mechanism of [4Fe-4S] cluster assembly exists and is operational in *S. salmonicida* as well as in some of the CL3 clade species.

These *in silico* analyses indicate that like in other fornicates, including *G. intestinalis* with its highly reduced mitosomes (Leger et al., 2019; Tachezy and Šmíd, 2019), one of the apparent reasons for having MROs retained in the CL3 lineage is their role in supplying the cells with Fe-S clusters critical for the function of various proteins (in cellular compartments beyond the MRO itself). Particularly notable is the presence of homologs of the mitochondrial import machinery and ISC pathway components in *I. spirale*, which provides strong evidence that this organism does possess a MRO, despite the fact that it has not yet been discerned in TEM preparations (Yubuki et al., 2017). Such a discrepancy between genomic analyses and ultrastructural observations concerning the presence of a MRO is not unprecedented, as has been noted in the case of the parasitic rhizarian *Mikrocytos mackini* (Burki et al., 2013) and *Retortamonas* spp. (Füssy et al., 2021).

### The Evolutionary Path of Mitochondrion-Related Organelle Reduction in the CL3 Lineage

**FIGURE 5:**
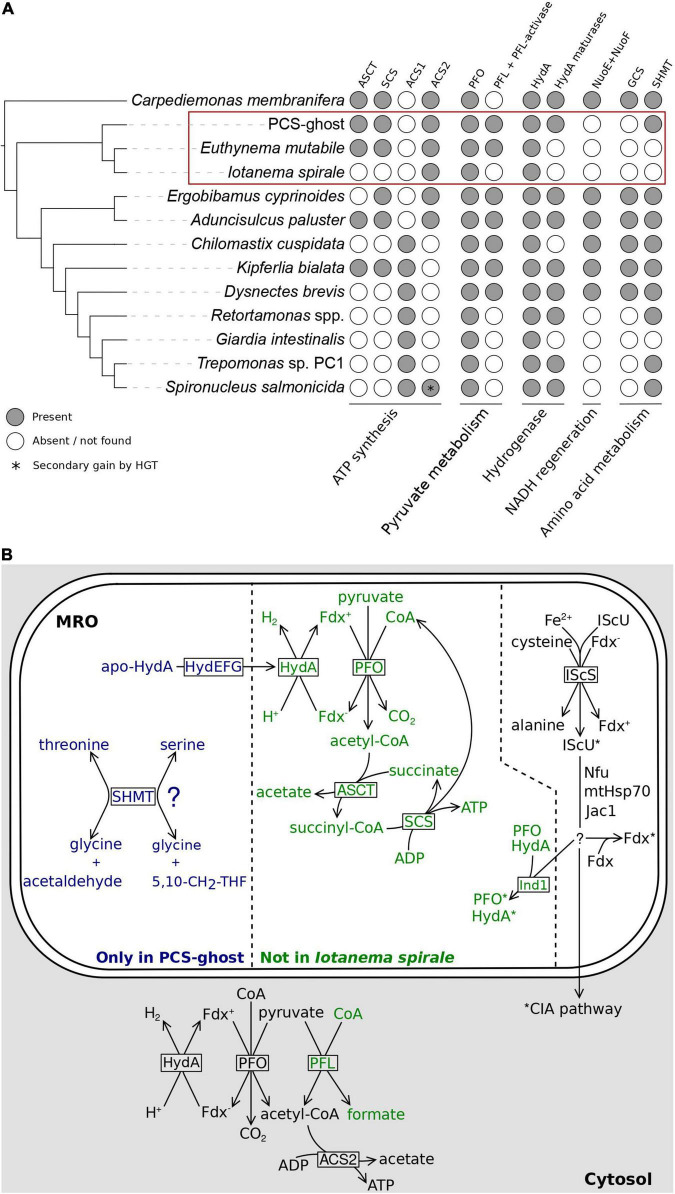
Metabolic functions of the MRO in the CL3 clade. **(A)** Occurrence of homologs of metabolic enzymes potentially linked to the MRO in CL3 clade members and other selected metamonads. Details on the genesis of the figure are as stated in the Figure 4 legend. **(B)** Partial reconstructions of MROs in three different representatives of the fornicate CL3 clade and relevant cytosolic reactions discussed in main text. The division of the schematically rendered MRO into three sectors reflects three subsets of inferred MRO-associated components differing in their distribution: shared by *Iotanema spirale*, *Euthynema mutabile*, and PCS-ghost (right); present only in the latter two (middle); present only in PCS-ghost (left). Protein factors mediating (catalyzing) the different reactions are shown in boxes at the respective reaction arrows. Fe-S clusters are indicated by asterisks at the proteins carrying them. Details of the Fe-S cluster assembly pathway could not be reconstructed with confidence and the scheme provided in the figure (including the specific role of Ind in the transfer of the clusters to HydA and PFO) must be treated as incomplete and speculative. The question mark at SHMT indicates the uncertainty regarding the actual reaction catalyzed by this enzyme in the PCS-ghost MRO (see main text). PFL and the respective reaction is rendered in green, as it is missing from *I. spirale*. Abbreviations: 5,10-CH_2_-THF, 5,10-methylene-tetrahydrofolate; apo-HydA, HydA precursor lacking the H-cluster important for catalytic activity; ASCT, acetate: succinyl-CoA transferase; Fdx, [2Fe-2S]ferredoxin (reduced and oxidized form distinguished by the minus and plus sign in the superscript); HydA, [FeFe]-hydrogenase; HydEFG, hydrogenase maturases (separate proteins HydE, HydF, and HydG); MPP, mitochondrial processing peptidase (subunits alpha and beta); PFL, pyruvate formate lyase; PFO, pyruvate:ferredoxin oxidoreductase; SCS, succinyl-CoA synthetase; SHMT, serine hydroxymethyltransferase.

To illuminate the range of physiological functions of the MROs in the CL3 lineage representatives beyond the assembly of Fe-S clusters, we searched their transcriptome assemblies for homologs of components of various metabolic pathways previously demonstrated or hypothesized to be localized to MROs in other metamonads (Leger et al., 2017; Yazaki et al., 2020; Füssy et al., 2021; Stairs et al., 2021). The sets of homologs recovered for the three species decreased in their complexity in the order PCS-ghost > *E. mutabile* > *I. spirale* (Figure 5A and Supplementary Table 3), which is in contrast to the similarity in the complement of MRO protein import and ISC pathway components in these organisms. As we argue below, the results of these analyses support a notion that different CL3 members exhibit different MRO types.

Most notable is the presence in PCS-ghost and *E. mutabile*, but not in *I. spirale*, of acetate: succinyl-CoA transferase (ASCT; also called succinyl-CoA:acetate CoA-transferase) and of both subunits (alpha and beta) of succinyl-CoA synthetase (SCS). The former enzyme is of the ACT1B type in both species, as seen in other groups of fornicates that possess ASCT (Leger et al., 2017). ASCT and SCS together participate in a pathway that enables the utilization of acetyl-CoA for ATP synthesis (Figure 5B). When present, these two enzymes are invariably localized to mitochondria and MROs (Leger et al., 2017), which is consistent with the predicted MRO targeting of most of these proteins from PCS-ghost and *E. mutabile* (Supplementary Table 3). However, this is apparently not the only route of ATP generation by substrate-level phosphorylation from acetyl-CoA in these two species, as they also possess the enzyme acetyl-CoA synthetase, specifically ACS2, which is also found in *I. spirale* (Supplementary Table 3). The presence of ACS2 in the CL3 lineage is consistent with its phylogenetic position within fornicates, i.e., its branching prior to the divergence of the “ACS1 group,” which is postulated to have replaced the ancestrally present ACS2 enzyme with a newly acquired ACS1 form (Leger et al., 2017; Yazaki et al., 2020). It was previously concluded that ACS2 is most likely a cytosolic enzyme in fornicates and their sister group barthelonids (Leger et al., 2017; Yazaki et al., 2020; Füssy et al., 2021), and indeed, ACS2 proteins in the CL3 representatives investigated are not predicted to localize to the MRO (Supplementary Table 3). These analyses indicate that the MROs in PCS-ghost and *E. mutabile* are energetic organelles, i.e., produce ATP by substrate-level phosphorylation (with acetate as a waste product), presumably contributing to the overall cellular ATP pool in parallel to cytosolic ATP sources (Figure 5B). In contrast, no evidence points to the *I. spirale* MRO playing a role in energy production, although genome sequencing will be required to confirm the absence of enzymes involved in MRO-localized ATP synthesis.

A question then arises: what is the source of acetyl-CoA utilized by the MROs of PCS-ghost and *E. mutabile*? Conventional mitochondria generate acetyl-CoA from pyruvate by the action of pyruvate dehydrogenase, which is typically missing from anaerobic MROs (Stairs et al., 2015; Gawryluk and Stairs, 2021) and we found no evidence for it in any of the three CL3 lineage representatives analyzed. Three other enzymes can catalyze the conversion of pyruvate to acetyl-CoA and are characteristically associated with obligate or facultative anaerobiosis: pyruvate:ferredoxin oxidoreductase (PFO), pyruvate:NADP^+^ oxidoreductase (PNO), and pyruvate formate lyase (PFL; Stairs et al., 2015; Gawryluk and Stairs, 2021). PFO has previously been reported from various Fornicata representatives, including ones without acetyl-CoA utilization in MROs (Stairs et al., 2015; Hamann et al., 2017; Tanifuji et al., 2018; Füssy et al., 2021), so it was not surprising to find PFO in all three CL3 lineage members (Supplementary Table 3). In fact, multiple PFO isoforms are present in each species, and they fall into three different clades in a broader PFO phylogeny (Supplementary Figure 8). One of the PFO subgroups from CL3 species includes both PFO isoforms found in the transcriptome assembly from *I. spirale* and is part of a clade (denoted here group A) that also includes PFOs from diplomonads that were experimentally demonstrated to be cytosolic (Jerlström-Hultqvist et al., 2013). In accordance with this, none of these CL3 PFOs are predicted to localize to the MRO (Supplementary Table 3). The other two PFO subgroups in CL3 organisms are restricted to PCS-ghost and *E. mutabile* and each belongs to a broader clade (group B and group C, respectively) comprising PFOs from other free-living fornicates (Supplementary Figure 8). Importantly, both PCS-ghost and *E. mutabile* have at least one of these PFOs predicted to be MRO-localized (Supplementary Table 3).

There was no evidence for PNO in the CL3 lineage or in any other metamonad queried, but our searches revealed the presence of another pyruvate-converting enzyme, PFL, in PCS-ghost and *E. mutabile* (but not *I. spirale*; Supplementary Table 3). Although PFL had not been reported from any other metamonad, we searched available sequence data sets and identified PFL in five other fornicates and *Barthelona* sp. PAP020 (Supplementary Table 5). The presence of a functional PFL enzyme in all of these species is supported by the fact that they also encode a homolog of the specific PFL-activating enzyme (PFL-AE; Supplementary Tables 3, 5; Stairs et al., 2011). Interestingly, the PFL sequence from *Barthelona* does not cluster with PFL proteins from fornicates but is instead related to PFL from a distantly related eukaryotic anaerobe, the breviate *Pygsuia biforma* (Supplementary Figure 9). This suggests that PFL was acquired independently by different metamonad lineages, consistent with the previous insights concerning the role of horizontal gene transfer in disseminating the PFL gene among eukaryotes (Stairs et al., 2011). According to the subcellular targeting predictions, PFL in both PCS-ghost and *E. mutabile* is most likely cytosolic. Combined, our analyses suggest that PCS-ghost and *E. mutabile* convert pyruvate to acetyl-CoA both in the cytoplasm and in the MRO, whereas only the cytoplasmic route is present in *I. spirale* (Figure 5B).

Reduced ferredoxin resulting from the reaction of PFO needs to be reoxidized, which in MROs is typically achieved by passing the electrons to protons in a reaction catalyzed by [FeFe]-hydrogenase (HydA), yielding H_2_ (Stairs et al., 2015). Metamonads commonly possess multiple HydA versions, with their predicted or experimentally demonstrated localization to either MROs or the cytoplasm (Jerlström-Hultqvist et al., 2013; Leger et al., 2017; Stairs et al., 2021; Smutná et al., 2022). Unsurprisingly, each CL3 lineage representative encodes multiple HydA isoforms, but only one, a protein from *E. mutabile*, was predicted to be MRO-localized (Supplementary Table 3). According to our phylogenetic analysis, this HydA protein has orthologs in PCS-ghost and several other fornicates (but not *I. spirale*), with at least one of them (the *C. membranifera* protein, the only other fornicate homolog with a complete N-terminus) also predicted to be MRO-localized (see Leger et al., 2017; the same conclusion reached here by using NommPred). Crucially, PCS-ghost encodes a full set of hydrogenase maturation factors (HydE to HydG), which are considered typical MRO proteins based on experimental data and bioinformatic predictions (Stairs et al., 2015, 2021; Leger et al., 2017). Indeed, at least two of the maturation factors in PCS-ghost have a predicted MRO localization (Supplementary Table 3). As no hydrogenase-independent function of these proteins is known, their presence in PCS-ghost supports the notion that there is a hydrogenase present in the mitochondrial organelle of this organism that is not recognized as such by the prediction programs employed or was missed in the transcriptome data obtained.

Somewhat surprisingly, while there is a predicted candidate for a MRO-localized hydrogenase in *E. mutabile*, none of the hydrogenase maturation factors were identified in its transcriptome assembly. We cannot completely rule out the possibility that the maturation factors are encoded, but with an expression level too low (at the conditions used for obtaining the transcriptome data) to detect their presence. Nevertheless, the absence of the maturation factors in several hydrogenase-carrying eukaryotes has been noted before (Stairs et al., 2015; Leger et al., 2017; Yazaki et al., 2020) and there is growing biochemical evidence that hydrogenases can attain enzymatic activity without the aid of these factors (Berto et al., 2011; Kuchenreuther et al., 2012; Smutná et al., 2022). Hence, despite various uncertainties inherent to the nature of the data analyzed and the solely *in silico* methodology employed, the results of our examinations are best interpreted such that the MROs of both PCS-ghost and *E. mutabile* contain a functional hydrogenase enzyme (Figure 5B), consistent with the inferred presence of PFO (and thus a source of reduced ferredoxin) in the same compartments. In contrast, we see no evidence for the presence of a hydrogenase in the MRO of *I. spirale*, which is in accord with the apparent lack of PFO in the organelle.

In contrast to many other fornicates (Leger et al., 2017), none of the CL3 lineage representatives were found to encode NuoE and NuoF proteins. In *Trichomonas vaginalis* these proteins interact with HydA to couple energetically favorable oxidation of reduced ferredoxin (Fdx^–^) to unfavorable oxidation of NADH (Dyall et al., 2004) and are thought to function analogously in fornicate MROs to regenerate NAD^+^ in the organelle (Leger et al., 2017). The absence of these proteins in the CL3 lineage representatives may relate to the fact that they also lack homologs of the subunits of the glycine cleavage system (GCS), a standard mitochondrial module present in MROs of various metamonads that generates NADH as part of its activity (Zubáčová et al., 2013; Leger et al., 2017; Stairs et al., 2021). Thus, it is possible that without the GCS, there is no source of NADH in the MROs of the CL3 lineage members and hence no need for a route of NADH reoxidation within the organelle. It was previously speculated that the loss of the GCS observed in some metamonads as well as other eukaryotes is related to a transition to parasitism (Leger et al., 2017). The only previously reported free-living eukaryote devoid of the GCS is the diplomonad *Trepomonas* sp. PC1 (Leger et al., 2017), which was hypothesized to have reverted to a free-living life-style secondarily from an endobiotic ancestor (Xu et al., 2016). There is no evidence for PCS-ghost or *E. mutabile* to be secondarily free-living, which suggests that the loss of the GCS is not strictly linked to the acquisition of a parasitic (or generally endobiotic) lifestyle.

Interestingly, the lack of the GCS (as an obvious MRO-localized source of NADH) and NuoE/NuoF (as part of the NADH reoxidation mechanism) in the CL3 lineage provides additional, though indirect, evidence for our hypothesis that the MRO in *E. mutabile* and PCS-ghost houses the PFO enzyme. We note that previous authors reporting on fornicate pyruvate conversion enzymes were hesitant to speculate on their subcellular localization without experimental data (Leger et al., 2017, 2019), and we agree that the experimentally studied diplomonad *S. salmonicida* is so far the only fornicate in which the presence of PFO in its MRO has been robustly established (Jerlström-Hultqvist et al., 2013). It is indeed conceivable that PFO is generally missing from fornicate MROs despite the fact that most of them are thought to possess a MRO-localized hydrogenase, as the latter would still be required for NuoE/NuoF-assisted NADH reoxidation. However, given the apparent absence of this process in *E. mutabile* and PCS-ghost, the existence of a MRO-localized hydrogenase postulated for both organisms (see above) calls for another organellar process producing a reduced substrate for the enzyme. Analogous to well-studied metamonad hydrogenosomes (*S. salmonicida* and *T. vaginalis*), the activity of PFO generating Fdx^–^ as a substrate for hydrogenase-mediated reoxidation is the most obvious candidate (Figure 5B).

Functionally related to the GCS is the enzyme serine hydroxymethyltransferase (SHMT), which utilizes one of the products of the GCS activity, 5,10-methylene-tetrahydrofolate (5,10-CH_2_-THF), to synthesize serine in the mitochondrion and in MROs (Leger et al., 2017). While *E. mutabile* and *I. spirale* appear to lack this enzyme, PCS-ghost has it (Supplementary Table 3). The presence of SHMT in the absence of the GCS is not unprecedented, as this was previously observed in several other metamonads (Leger et al., 2017; Füssy et al., 2021). It is thus conceivable that SHMT in these organisms is used to catalyze the cleavage of serine to glycine to produce 5,10-CH_2_-THF, a source of one-carbon units for various biosynthetic reactions, such as the production of thymidylate or methionine (Ducker and Rabinowitz, 2017). However, apart SHMT we did not find any other candidate for an enzyme utilizing or producing 5,10-CH_2_-THF in PCS-ghost, a situation previously encountered in *Retortamonas* spp. (Füssy et al., 2021). It is thus possible that SHMT functions in these organisms in a tetrahydrofolate-independent manner as an aldolase cleaving β-hydroxyamino acids into glycine and corresponding aldehydes (Figure 5B), as has been demonstrated for SHMT from several other organisms (Chiba et al., 2012).

## Conclusion

In this report we characterized and formally described a free-living relative of *I. spirale* and (presumably) *C. mobilis*, two endobiotic fornicates united into the family Caviomonadidae. Our results demonstrate that the simplification of the mastigont characteristic for caviomonads evolved independently of the endobiotic lifestyle and support the inclusion of free-living organisms like *E. mutabile*, and potentially also PCS-ghost, into this family. By obtaining transcriptome data from *E. mutabile* and an unidentified relative of caviomonads we pinpointed the origin of the UAG codon reassignment previously documented from *I. spirale* and for the first time addressed the phylogenetic position of the fornicate CL3 clade using a phylotranscriptomic approach. While our analyses place the CL3 clade as one of the earliest diverging fornicate lineages, they also cast doubt on the authenticity of some of the sequence data previously attributed to *H. teleskopos* and suggest that the previous conclusions on its position in the fornicate phylogeny may be misleading. Establishing a new culture of this or a closely related fornicate and obtaining representative genome-level data is thus an obvious prerequisite for a more accurate and comprehensive understanding of fornicate diversity and evolution.

Most importantly, our analyses of the previously available and newly obtained transcriptome data enabled us to reconstruct the outlines of the evolutionary history of the mitochondrion in the CL3 lineage and to document the existence of a cryptic mitochondrial remnant in *I. spirale*. Specifically, we inferred that like fornicates as a whole, the CL3 lineage was ancestrally endowed with a MRO form that can be classified as a hydrogenosome, owing to the fact that it is predicted to be a place of both H_2_ and ATP production. The MRO in *E. mutabile* seems to still conform to the definition of a hydrogenosome, despite the loss of several components (hydrogenase maturation factors, SHMT) retained by at least some other extant CL3 lineage representatives (i.e., PCS-ghost). In contrast, no evidence for the production of H_2_ and ATP by the cryptic MRO in *I. spirale* could be found by our analyses of transcriptome data from this organism, suggesting that the organelle conforms to the definition of a mitosome. Although further studies, including analyses of genome sequences of *I. spirale* and other caviomonads are required to corroborate these conclusions, the data at hand indicate that the evolutionary history of the CL3 lineage presents a separate case of hydrogenosome-to-mitosome transition in metamonads, independent from the one previously documented for the lineage leading to *Giardia*. It is interesting to note that both transitions seem to correlate with adopting an endobiotic lifestyle by the respective fornicate lineages, which may suggest that similar changes in or relaxation of selection forces underlie the MRO sculpting in both groups. Finally, our investigations further cement the notion that oxymonads may be the sole eukaryote group that has managed to lose the mitochondrion.

## Taxonomic Summary

Taxonomic assignment: Eukaryota: Metamonada: Fornicata: Caviomonadidae Cavalier-Smith, 2013.

ZooBank number of this work: urn:lsid:zoobank.org: pub:AB72A6DE-1C47-45BA-9C0A-13C773F47B68.

### *Euthynema* Hanousková & Čepička, gen. nov.

Diagnosis: Free-living Caviomonadidae with non-helical cells. Four basal bodies in the flagellar apparatus. The mitochondrion present in the form of a hydrogenosome. Type and only species: *Euthynema mutabile* sp. nov. Etymology: *euthy-* (from Gr. εu’ θúς, straight) + L. noun *nema* (thread). Neuter gender.

### *Euthynema mutabile* Hanousková & Čepička, sp. nov.

Diagnosis: as for the genus *Euthynema*. Holotype: Protargol-stained cell of the strain PCSc10 depicted in Figure 1J. The slide with the holotype is deposited in the collection of the National Museum in Prague, Czech Republic, inventory number P6E 5299. Type locality: Prince Cove, MA, United States (41°38′N, 70°24′W). Habitat: Littoral anoxic sediments. Etymology: L. adj. *mutabile* (changeable).

## Data Availability Statement

Illumina RNAseq reads generated in this study and the transcriptome assembly for the PCSc10 isolate (=*E*. *mutabile*) are available at the National Center for Biotechnology Information (NCBI) under BioProject accession PRJNA812385. Transcriptome assembly-derived rRNA sequences from *I. spirale*, *E. mutabile*, and PCS ghost were deposited at GenBank with accession numbers OM987995–OM987997. The assembly of subtracted RNAseq data corresponding to PCS ghost, multiple sequence alignments, and original phylogenetic trees were deposited at figshare (https://figshare.com/projects/Evidence_for_an_independent_hydrogenosome-to-mitosome_transition_in_the_CL3_lineage_of_fornicates/134543).

## Author Contributions

RV carried out computational reconstructions of MROs. PH performed the morphological and ultrastructural characterization of *E. mutabile* and isolated RNA for transcriptome sequencing. JS carried out the phylogenomic analysis with PhyloFisher, some of the single-gene phylogenetic analyses, and prepared some of the figures. JDS isolated the PCS culture and performed its initial characterization. DŽ generated the transcriptome assemblies and evaluated the genetic code. ME conceived the study, supervised RV, JS, and DŽ, and drafted the manuscript. IČ conceived the study, supervised PH, and performed the 18S rRNA phylogeny. All authors contributed to the writing and approved the final version of the manuscript.

## Conflict of Interest

The authors declare that the research was conducted in the absence of any commercial or financial relationships that could be construed as a potential conflict of interest.

## Publisher’s Note

All claims expressed in this article are solely those of the authors and do not necessarily represent those of their affiliated organizations, or those of the publisher, the editors and the reviewers. Any product that may be evaluated in this article, or claim that may be made by its manufacturer, is not guaranteed or endorsed by the publisher.

## Footnotes

1
https://www.atcc.org/-/media/product-assets/documents/microbial-media-formulations/atcc-medium-1525.pdf?rev=3b91106066af4916819c28239f7643e6

2
http://sbmorphometrics.org/soft-dataacq.html

3
https://mafft.cbrc.jp/alignment/server/index.html

4
https://itol.embl.de/

5
http://tree.bio.ed.ac.uk/software/figtree/

6
https://inkscape.org/

7
http://pfam.xfam.org/

8
https://services.healthtech.dtu.dk/service.php?TargetP-2.0

9
http://mitf.cbrc.jp/MitoFates/cgi-bin/top.cgi
