# Supplementary material for: Evidence for an Independent Hydrogenosome-to-Mitosome Transition in the CL3 Lineage of Fornicates
Source: Front Microbiol. 2022 May 19;13:866459. doi: 10.3389/fmicb.2022.866459 (PMC9161772; doi:10.3389/fmicb.2022.866459)
Supplement: Supplementary file 1 [file Data_Sheet_1.PDF]

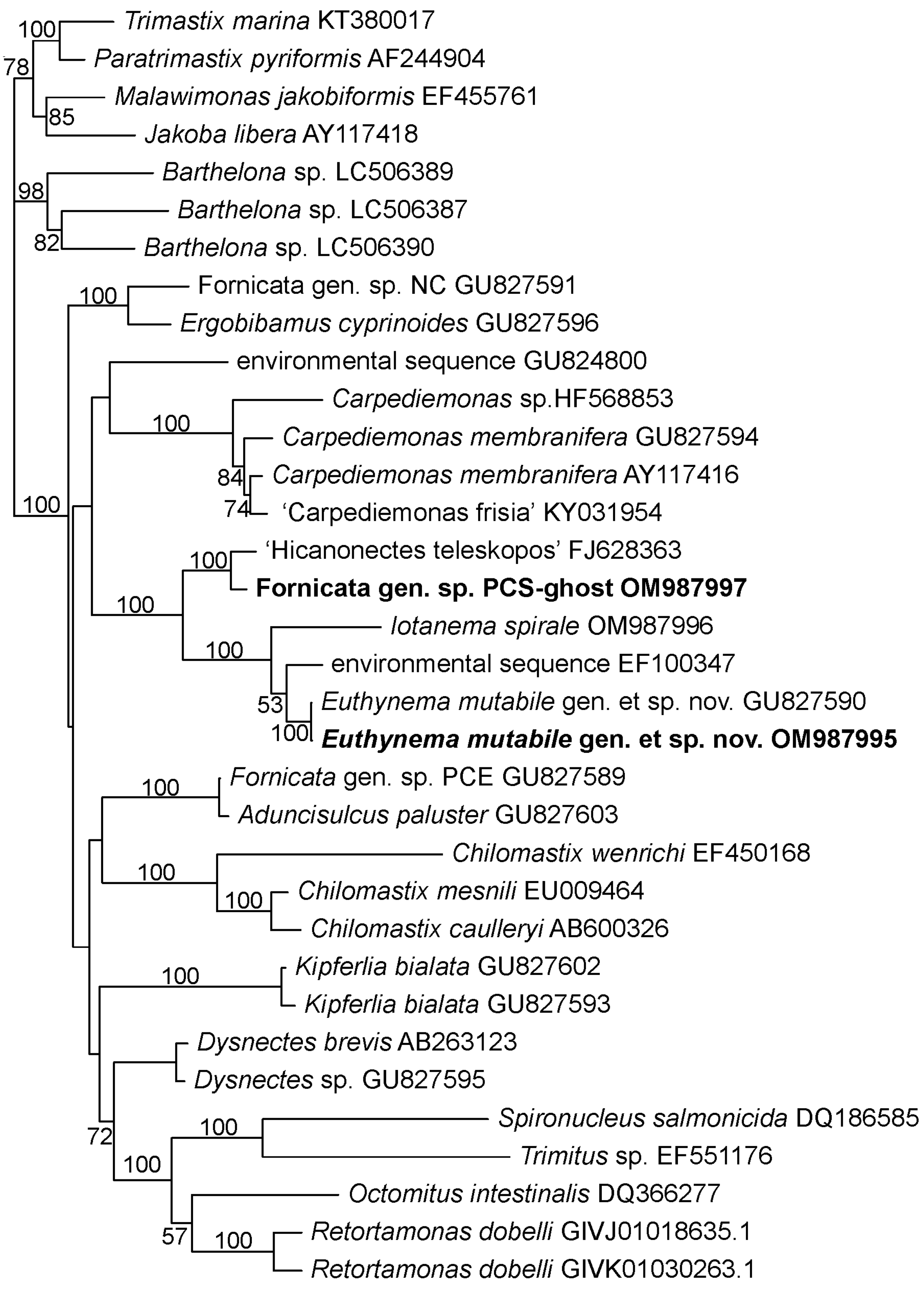

Figure S1. Phylogenetic position of *Euthynema mutabile* and PCS-ghost based on 18S rRNA gene sequences. The tree was inferred using maximum likelihood in RAxML under the GTRGAMMAI substitution model. Values at branches correspond to bootstrap support values calculated from 1000 pseudoreplicates. Sequences in bold were newly generated in this study. Quotation marks indicate organismal names that are used in the literature but have not been validly described ('Carpediemonas frisia') or point to the uncertainty regarding the origin of the respective 18S rRNA gene sequence ('Hicanonectes teleskopos').

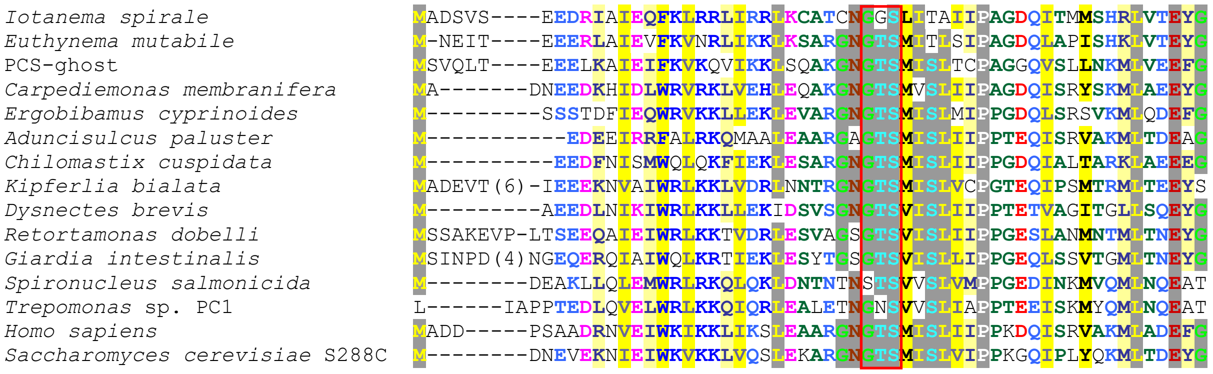

Figure S2. Multiple sequence alignment of the N-terminal segment of eRF1 proteins from fornicates (with sequences from *H. sapiens* and *S. cerevisiae* included as a well-studied reference) including the conserved GTS motif (boxed in red) implicated in termination codon binding. Note the unique mutation in the motif in the *I. spirale* eRF1 not shared by homologs from the closely related taxa, i.e. *E. mutabile* and PCS-ghost (unidentified CL3 clade representative), correlating thus with the stop-to-Gln reassignment of the UAG codon (see main text). The motif is also altered in eRF1 sequences from the two representatives of the diplomonad subgroup Hexamitinae included in the alignment (*S. salmonicida*, *Trepomonas* sp. PC1), which, like *I. spirale*, have a variant genetic code (stop-to-Gln reassignment of both UAG and UAA). The alignment was processed by using CHROMA Version 1.0 (http://www.llew.org.uk/chroma/). Accession numbers or IDs of the sequences included in the alignment: *Iotanema spirale* – GFCE01001756.1; *Euthynema mutabile* – TRINITY_DN17831_c0_g1_i1; PCS-ghost – TRINITY_DN43119_c0_g1_i1; *Carpediemonas membranifera* – KAG9391744.1; *Ergobibamus cyprinoides* – gnl|Ergobibamus_cyprinoides|766; *Aduncisulcus paluster* – gnl|Carplike_NY0171|3326_a4478;630; *Chilomastix cuspidata* – gnl|Chilomastix_cuspidata|287; *Kipferlia bialata* – GIQ85797.1; *Dysnectes brevis* – gnl|Dysnectes_brevis|239_a270;2921; *Retortamonas dobelli* – GIVJ01019945.1; *Giardia intestinalis* – XP_001710000.1; *Spironucleus salmonicida* – KAH0576224.1; *Trepomonas* sp. PC1 – GDID01003271.1; *Homo sapiens* – NP_004721.1; *Saccharomyces cerevisiae* S288C – NP_009701.3

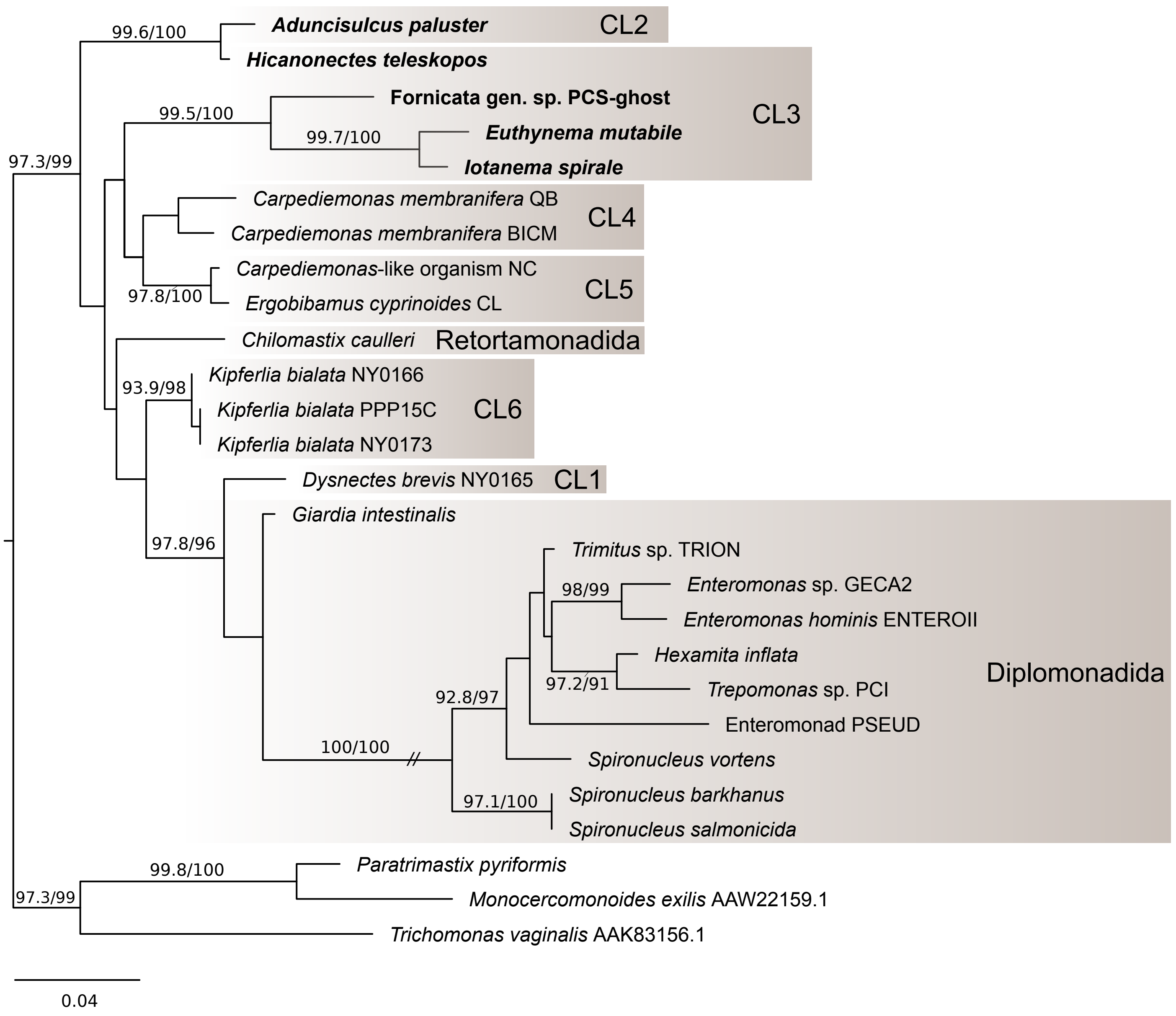

Figure S3. Phylogenetic analysis of α-tubulin sequences. The tree was inferred from a multiple alignment of amino acid sequences (366 positions) using IQ-TREE v2.1.2, with the substitution model (LG+G4) selected by the program. Branch support values were inferred with SH-aLRT / 10,000 ultrafast bootstrap pseudoreplicates and are shown when ≥ 80/95. Most of the sequences included in the analysis were adopted from a corresponding dataset used by Takishita et al. (2012), with the relevant updates in the names of some of the taxa (*Paratrimastix pyriformis*, formerly *Trimastix pyriformis*; *Aduncisulcus paluster*, formerly *Carpediemonas*-like organism NY0171). Newly added sequences are provided with their respective GenBank accession number. The root is placed arbitrarily between sequences from Fornicata and other metamonads. The assignment of individual sequences to the main lineages of Fornicata, including the various *Carpediemonas*-like (CL) clades defined by Kolisko et al. (2010), is indicated on the right. Note the paraphyly of the sequences corresponding to the clade CL3.

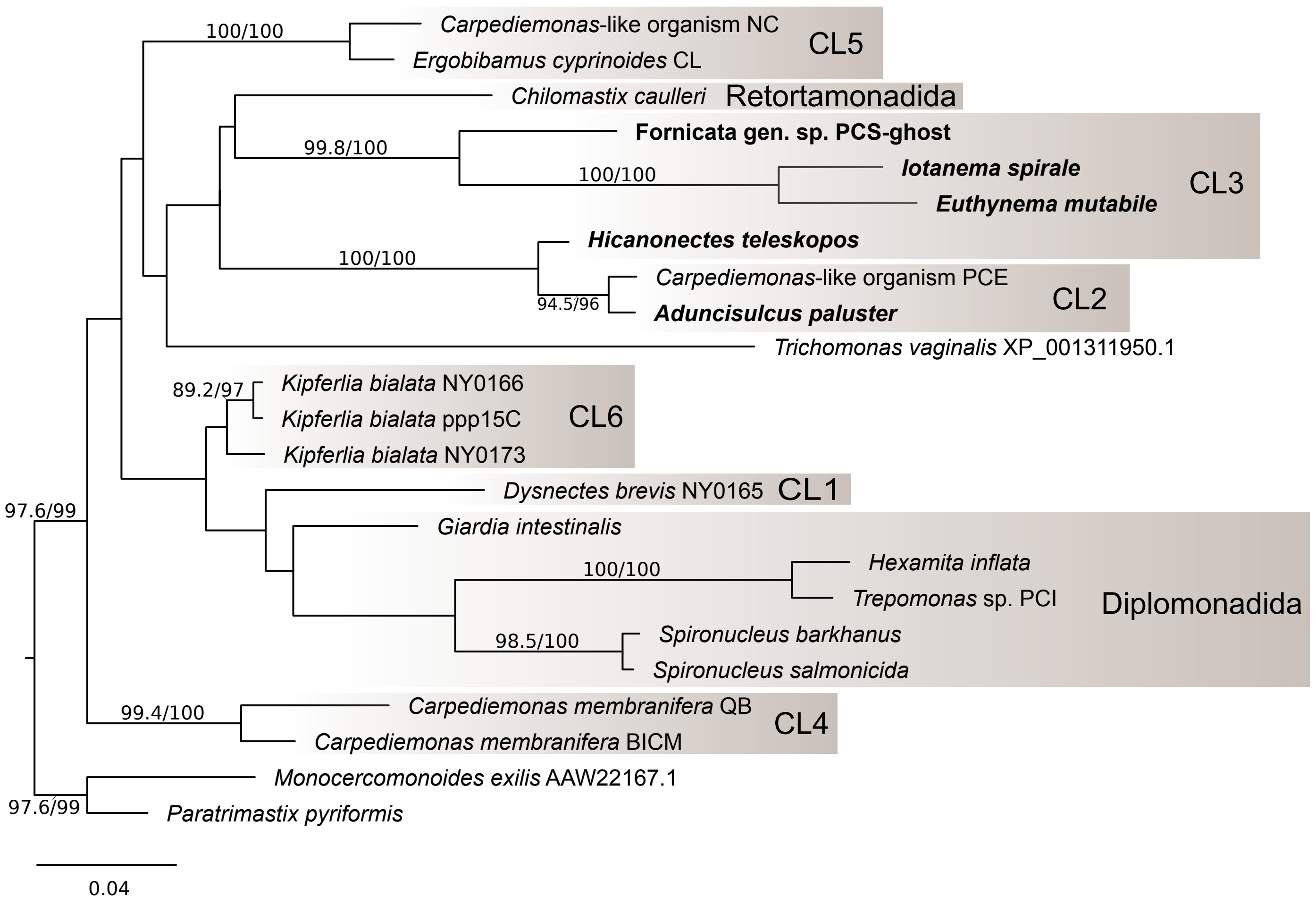

Figure S4. Phylogenetic analysis of β-tubulin sequences. For general explanation of the methodology of the tree inference and display conventions see the legend to Supplementary Figure S3. Details specific for this tree: the length of the alignment: 380 positions; substitution model: LG+G4. As in the α-tubulin tree, sequences from organisms assigned to the CL3 clade are paraphyletic.

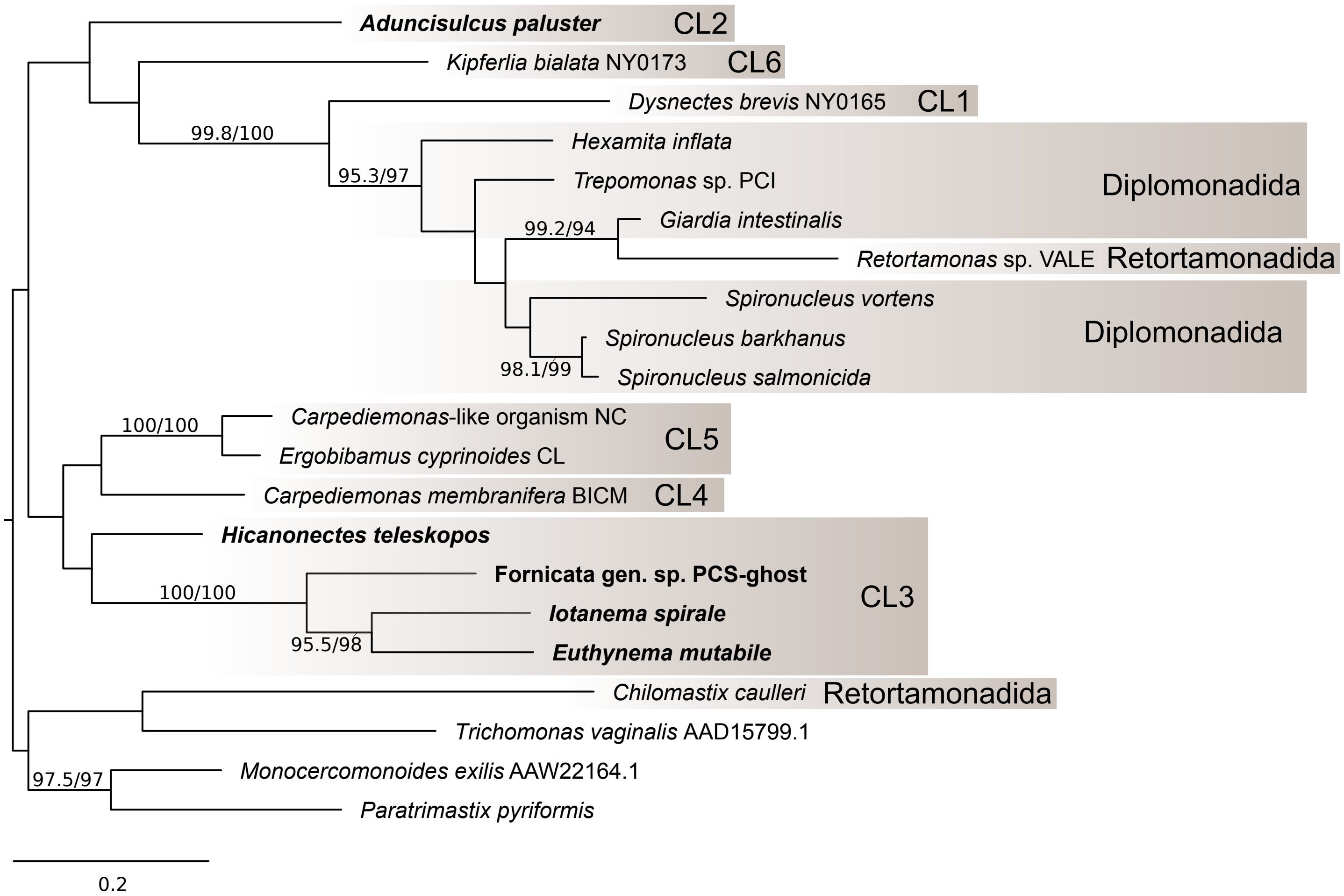

Figure S5. Phylogenetic analysis of EF1α sequences. For general explanation of the methodology of the tree inference and display conventions see the legend to Supplementary Figure S3. Details specific for this tree: 396 position; substitution model: LG+I+G4. Note that even though sequences from CL3 clade organisms do form a monophyletic group, the relative position of *Hicanonectes teleskopos* and PCS-ghost is in an obvious disagreement with the close relationship of the organisms indicated by the 18S rRNA gene phylogeny (Supplementary Figure S1).

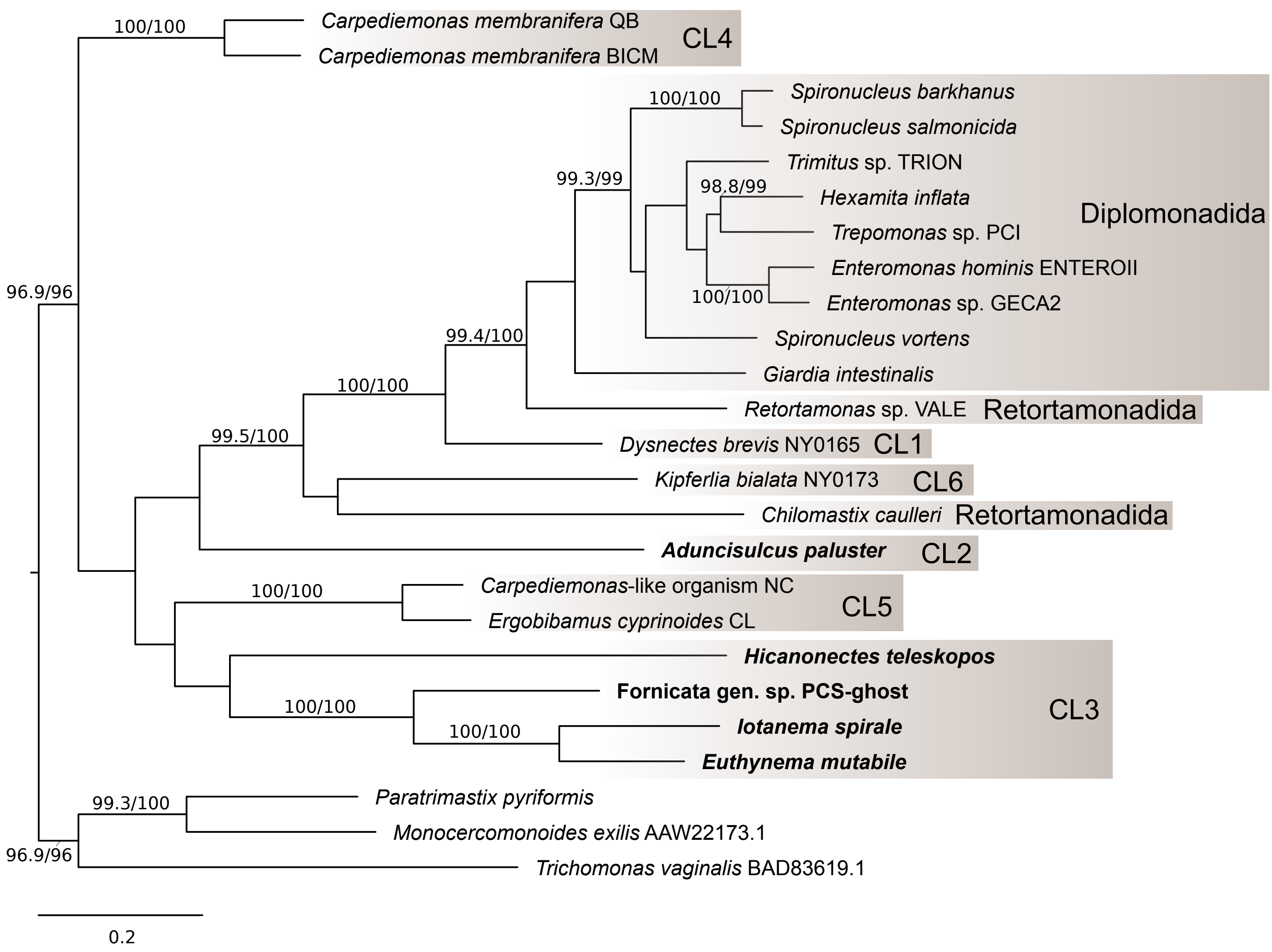

Figure S6. Phylogenetic analysis of HSP90 sequences. For general explanation of the methodology of the tree inference and display conventions see the legend to Supplementary Figure S3. Details specific for this tree: 674 positions; substitution model: LG+F+I+G4. As in the EF1α tree (Supplementary Figure S5), the relative position of *Hicanonectes teleskopos* and PCS-ghost is in an obvious disagreement with the close relationship of the organisms indicated by the 18S rRNA gene phylogeny (Supplementary Figure S1).

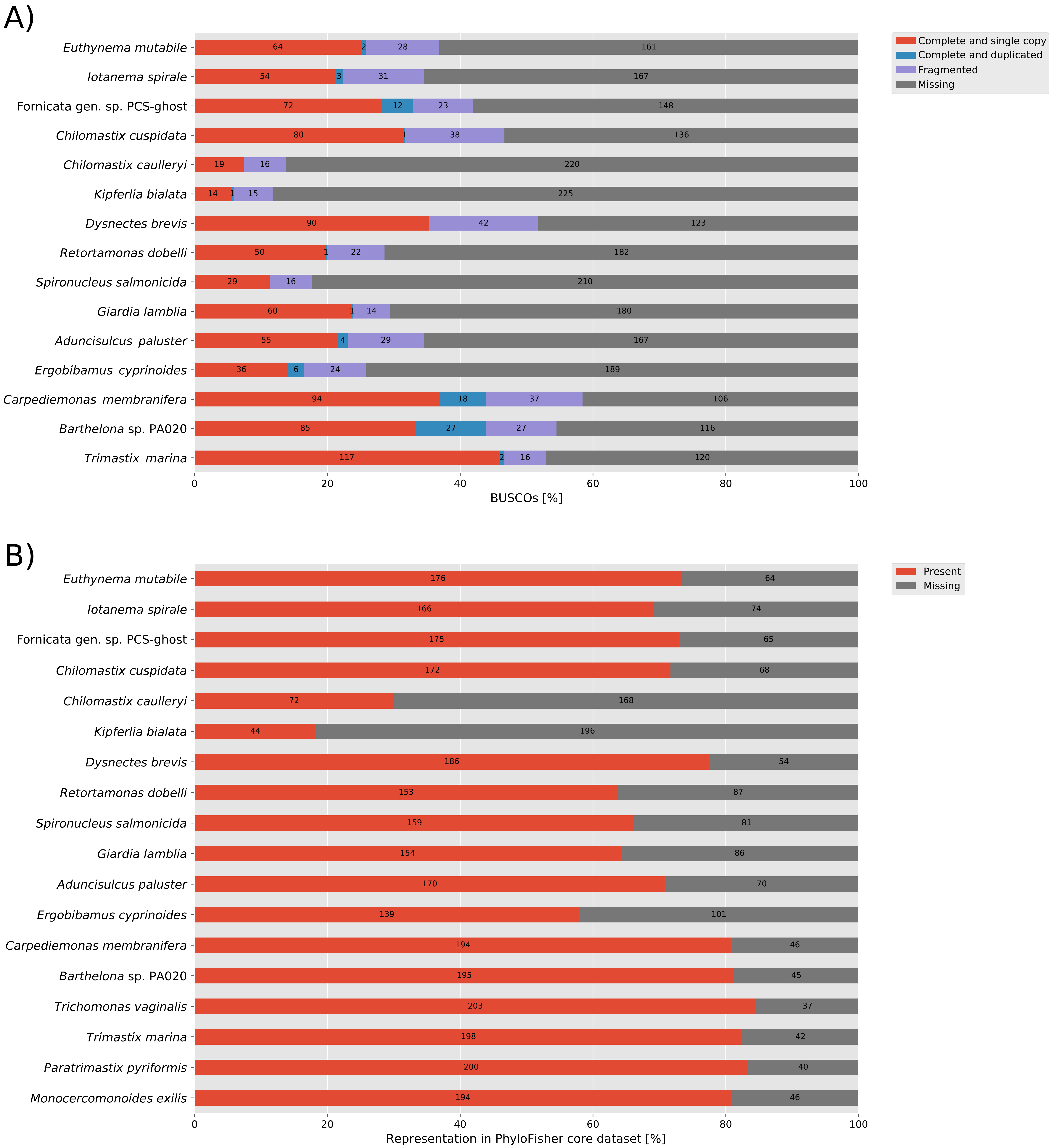

Figure S7. Assessment of the representativeness of the newly analyzed transcriptome assemblies. (A) Comparison to the conserved eukaryotic BUSCO gene set (comprised of 255 genes). Complete orthologs (either single-copy or duplicated) are ≥95% complete; fragmented orthologs are <95% complete; and missing orthologs are not found. (B) Comparison to the core eukaryote gene set included in the PhyloFisher database (comprised of 240 highly conserved genes). Numbers in the bars refer to the number of genes found/missing in the transcriptome assemblies.

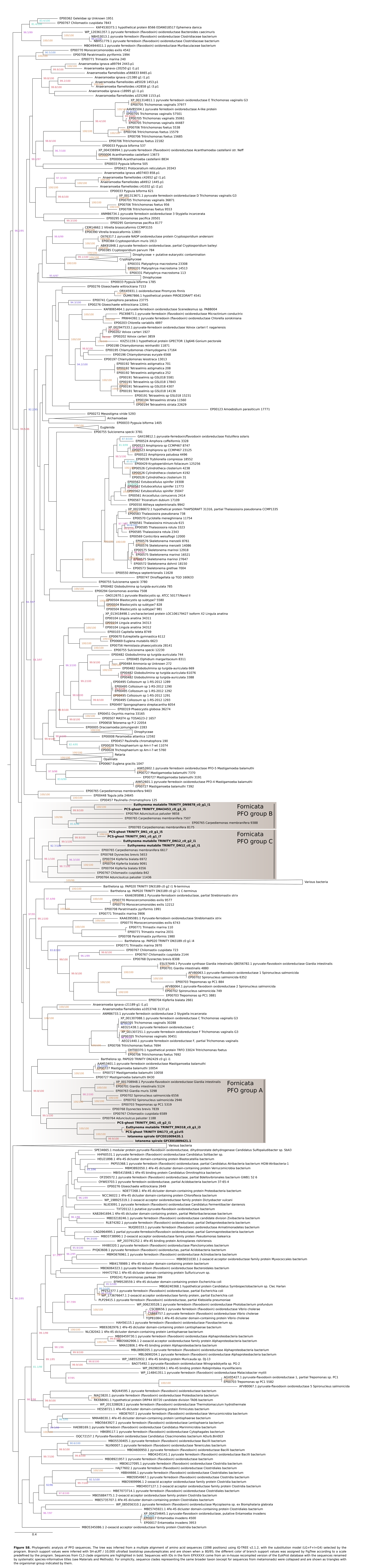

Figure S8. Phylogenetic analysis of PFO sequences. The tree was inferred from a multiple alignment of amino acid sequences (1098 positions) using IQ-TREE v2.1.2, with the substitution model (LG+F+I+G4) selected by the program. Branch support values were inferred with SH-aLRT / 10,000 ultrafast bootstrap pseudoreplicates and are shown when ≥ 80/95; the different color of branch support values was assigned by FigTree according to a scale predefined by the program. Sequences from CL3 clade organisms are highlighted in bold. Sequences with IDs in the form EPXXXXX come from an in-house recompiled version of the EukProt database with the sequences renamed by systematic species-informative titles (see Materials and Methods). For simplicity, sequence clades representing the same broader taxon (except for sequences from metamonads) were collapsed and are shown as triangles with the organismal group indicated by them.

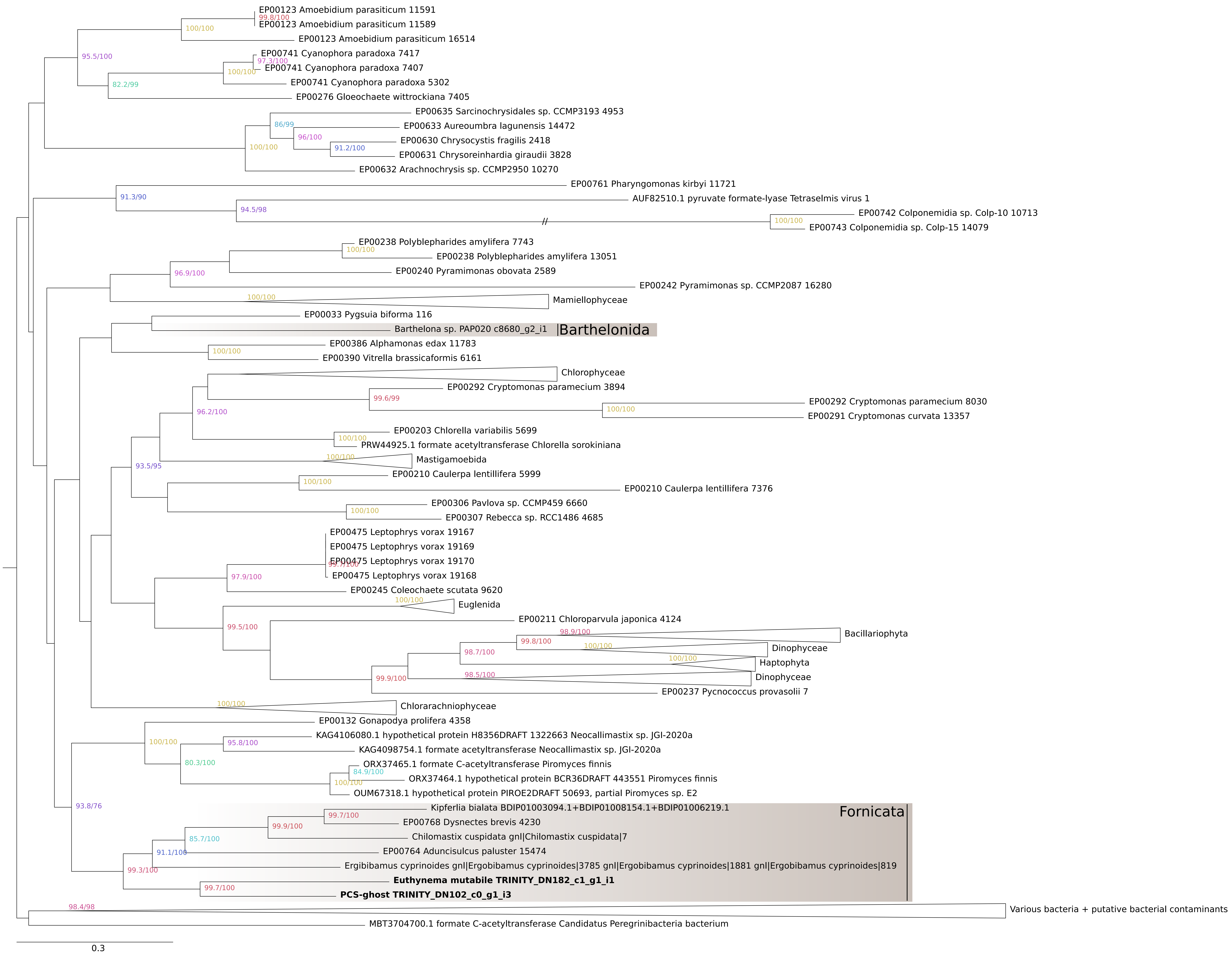

Figure S9. Phylogenetic analysis of PFL sequences. For general explanation of the methodology of the tree inference and display conventions see the legend to Supplementary Figure S8. Details specific for this tree: the length of the alignment: 712 positions; substitution model: LG+I+G4.

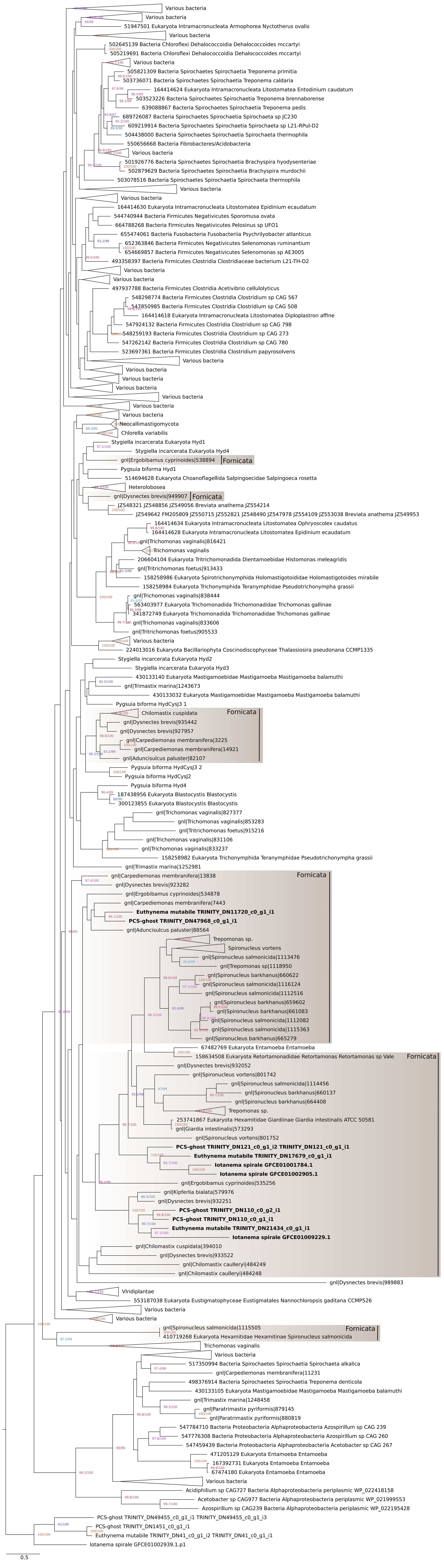

Figure S10. Phylogenetic analysis of HydA sequences. For general explanation of the methodology of the tree inference and display conventions see the legend to Supplementary Figure S8. Details specific for this tree: the length of the alignment: 554 positions; substitution model: LG+G4. The tree was rooted between bona fide putative hydrogenases and hydrogenase-related proteins of the Nar1 group.
