# Supplementary material for: Evidence for an Independent Hydrogenosome-to-Mitosome Transition in the CL3 Lineage of Fornicates
Source: Front Microbiol. 2022 May 19;13:866459. doi: 10.3389/fmicb.2022.866459 (PMC9161772; doi:10.3389/fmicb.2022.866459)
Supplement: Supplementary file 2 [file Data_Sheet_2.PDF]

Supplementary text S1: Suspicious origin of molecular marker sequences previously reported from *Hicanonectes teleskopos*

Hicanonectes teleskopos, a representative of the fornicate CL3 clade based on its 18S rRNA sequence, was robustly united with the CL2 clade (represented by two strains, one of them subsequently described as *Aduncisulcus paluster*) in the six and seven-gene phylogenies reported by Takishita et al. (2012). Digging into the details of the single-gene phylogenies presented by Takishita et al. (see their supplementary figures S1 to S7), the signal for a combined *Hicanonectes*-CL2 clade comes only from two genes, tubulins α and β, whereas in the trees inferred from the other genes present in both lineages, *Hicanonectes* and the CL2 lineage do not form a clade. Interestingly, the four- and five-gene phylogenies omitting the two tubulin genes did not recover a *Hicanonectes*-CL2 clade (supplementary figures S8 and S9 in Takishita et al., 2012), suggesting a gene-specific conflict in the phylogenetic signal. Indeed, in α- and β-tubulin phylogenies the sequences assigned to *H. teleskopos* (CL3) and the CL2 group form a very tight clade (reflecting very high similarity of the sequences), whereas no specific affinity of CL3 and CL2 sequences is recovered in the trees for the other two proteins (EF1α, HSP90) where such comparisons are possible (see supplementary figures S2, S3, S5, and S6 in Takishita et al., 2012).

To cast more light on this apparent discrepancy, we reanalyzed the phylogeny of the four protein-coding genes based on an expanded taxon sampling that now includes the respective homologs from caviomonads and PCS-ghost. In both tubulin phylogenies, the previously reported *H. teleskopos* sequences remained tightly clustered with the homologs from the CL2 lineage, whereas homologs from PCS-ghost and caviomonads formed a separate clade without a strongly supported relationship to any of the other fornicate lineage (Supplementary Figures 3 and 4). In the trees inferred from EF1α and HSP90 sequences, PCS-ghost and caviomonads likewise constituted a clade with full support, whereas *H. teleskopos* branched at the base of this clade, deeply diverged from the Caviomonadidae-PCS-ghost group and without strong statistical support for this particular position (Supplementary Figures 5 and 6). Hence, the different sequences presumably derived from *H. teleskopos* suggest three different possible phylogenetic positions of this organism within fornicates: (1) close to PCS-ghost, with both together sister to caviomonads (18S rRNA); (2) a separate lineage with a possible affinity to the Caviomonadidae-PCS-ghost clade (EF1α, HSP90); or (3) very close to *A. paluster* in the CL2 lineage (tubulins). Given the loss of the *H. teleskopos* culture we cannot check the authenticity of the sequences previously assigned to this organism, but we suspect they in fact do not all come from the same biological entity. Alternatively (but less likely), they may reflect a more complicated evolutionary history of the genes used as phylogenetic markers, such as unrecognized (hidden) paralogy or even horizontal gene transfer (see, e.g., Simpson et al., 2008).

There are, in fact, hints as to which of the makers may be more reliable and which should be treated with caution. The position of *H. teleskopos* suggested by the tubulin sequences seems to be at odds with the cytological features of this organism and *A. paluster*, as such a close relationship of the two species was not apparent from a detailed comparison of their cell morphology and ultrastructure (Yubuki et al., 2016). There is also reason to think that the position of *H. teleskopos* implied by the 18S rRNA sequence is suspicious. Specifically, the putative morphology of PCS-ghost (see above; Figure 1K-M) fits the characteristic and highly

derived morphology of caviomonads rather than that of the presumably closely related *H. teleskopos*, which has preserved a largely plesiomorphic fornicate (i.e., typical excavate) cell appearance (Park et al., 2009). Hence, two independent origins of caviomonad-like morphology or secondary reappearance of the plesiomorphic traits in the *H. teleskopos* lineage would have to be invoked if PCS-ghost and *H. teleskopos* are really sister taxa. Besides complicated gene histories, an alternative explanation is that only the EF1α and HSP90 sequences are authentic for *H. teleskopos*, whereas the 18S rRNA and tubulin sequences previously assigned to this organism come from two other fornicates, i.e. from a caviomonad relative and a putative CL2 member, respectively, that possibly contaminated the *H. teleskopos* culture (in a manner similar to PCS-ghost “contaminating” the original PCS culture).

References to Supplementary text

Park, J.S., Kolisko, M., Heiss, A.A., and Simpson, A.G. (2009). Light microscopic observations, ultrastructure, and molecular phylogeny of *Hicanonectes teleskopos* n. g., n. sp., a deep-branching relative of diplomonads. *J. Eukaryot. Microbiol.* 56, 373–384. doi: 10.1111/j.1550-7408.2009.00412.x

Simpson, A.G., Perley, T.A., and Lara, E. (2008). Lateral transfer of the gene for a widely used marker, alpha-tubulin, indicated by a multi-protein study of the phylogenetic position of Andalucia (Excavata). *Mol. Phylogenet. Evol.* 47, 366–377. doi: 10.1016/j.ympev.2007.11.035

Takishita, K., Kolisko, M., Komatsuzaki, H., Yabuki, A., Inagaki, Y., Cepicka, I., et al. (2012). Multigene phylogenies of diverse *Carpediemonas*-like organisms identify the closest relatives of 'amitochondriate' diplomonads and retortamonads. *Protist* 163, 344–355. doi: 10.1016/j.protis.2011.12.007

Yubuki, N., Huang, S.S., and Leander, B.S. (2016). Comparative Ultrastructure of Fornicate Excavates, Including a Novel Free-living Relative of Diplomonads*: Aduncisulcus paluster* gen. et sp. nov. *Protist* 167, 584–596. doi: 10.1016/j.protis.2016.10.001
